# Cerebral Microvascular Injury Induced by Lag3‐Dependent *α*‐Synuclein Fibril Endocytosis Exacerbates Cognitive Impairment in a Mouse Model of *α*‐Synucleinopathies

**DOI:** 10.1002/advs.202301903

**Published:** 2023-06-28

**Authors:** Qingxi Zhang, Qingrui Duan, Yuyuan Gao, Peikun He, Rui Huang, Haifeng Huang, Yanyi Li, Guixian Ma, Yuhu Zhang, Kun Nie, Lijuan Wang

**Affiliations:** ^1^ Department of Neurology Guangdong Neuroscience Institute Guangdong Provincial People's Hospital (Guangdong Academy of Medical Sciences) Southern Medical University Guangzhou 510080 China; ^2^ Guangdong Cardiovascular Institute Guangdong Provincial People's Hospital Guangdong Academy of Medical Sciences Guangzhou 510100 China; ^3^ Guangzhou Key Laboratory of Diagnosis and Treatment for Neurodegenerative Diseases Guangdong Provincial People's Hospital (Guangdong Academy of Medical Sciences) Southern Medical University Guangzhou 510080 China

**Keywords:** *α*‐Syn preformed fibril(s) (PFFs), *α*‐synucleinopathies, cerebral microvascular injury, cognitive impairment, lymphocyte‐activation gene 3 (Lag3)

## Abstract

The pathological accumulation of *α*‐synuclein (*α*‐Syn) and the transmission of misfolded *α*‐Syn underlie *α*‐synucleinopathies. Increased plasma *α*‐Syn levels are associated with cognitive impairment in Parkinson's disease, multiple system atrophy, and dementia with Lewy bodies, but it is still unknown whether the cognitive deficits in *α*‐synucleinopathies have a common vascular pathological origin. Here, it is reported that combined injection of *α*‐Syn preformed fibrils (PFFs) in the unilateral substantia nigra pars compacta, hippocampus, and cerebral cortex results in impaired spatial learning and memory abilities at 6 months post‐injection and that this cognitive decline is related to cerebral microvascular injury. Moreover, insoluble *α*‐Syn inclusions are found to form in primary mouse brain microvascular endothelial cells (BMVECs) through lymphocyte‐activation gene 3 (Lag3)‐dependent *α*‐Syn PFFs endocytosis, causing poly(ADP‐ribose)‐driven cell death and reducing the expression of tight junction proteins in BMVECs. Knockout of Lag3 in vitro prevents *α*‐Syn PFFs from entering BMVECs, thereby reducing the abovementioned response induced by *α*‐Syn PFFs. Deletion of endothelial cell‐specific Lag3 in vivo reverses the negative effects of *α*‐Syn PFFs on cerebral microvessels and cognitive function. In short, this study reveals the effectiveness of targeting Lag3 to block the spread of *α*‐Syn fibrils to endothelial cells in order to improve cognition.

## Graphical abstract

This work reveals that *α*‐synuclein (*α*‐Syn) preformed fibrils enter brain microvascular endothelial cells (BMVECs) via lymphocyte‐activation gene 3 (Lag3)‐dependent endocytosis, causing poly(ADP‐ribose)‐driven cell death of BMVECs, promoting *α*‐Syn reproduction in BMVECs and destroying the cerebral microvascular system, inducing the damage of cerebral microvessels, neurovascular unit coupling, and blood–brain barrier, then eventually leading to cognitive impairment in *α*‐synucleinopathies.

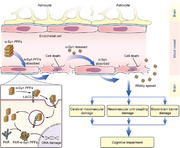

## 1 Introduction

*α*‐Synucleinopathies are a group of neurodegenerative diseases characterized by misfolding and aggregation of pathological *α*‐synuclein (*α*‐Syn) in damaged neurons or glial cells. This misfolding and aggregation of pathological *α*‐Syn results in the formation of insoluble *α*‐Syn inclusion bodies in diseases such as Parkinson's disease (PD), dementia with Lewy bodies (DLB), multiple system atrophy (MSA), the Lewy body variant of Alzheimer's disease, and pure autonomic failure.^[^ ^1^ ^]^ Pathological *α*‐Syn invades multiple neurotransmitter and body systems, inducing a wide range of motor and nonmotor clinical symptoms; the wide range of effects caused may be attributed to the heterogeneity of the *α*‐Syn protein itself, the different prion‐like spreading paths, and the different affected brain regions.^[^ ^2^ ^]^ Cognitive impairment is a main nonmotor symptom in *α*‐synucleinopathies and leads to a loss of self‐sufficiency in the late stage of disease, which is one of the most common causes of death.^[^ ^3^ ^]^

It is critical to study the possible common pathological origins of cognitive impairment in *α*‐synucleinopathies. *α*‐Syn oligomers exert neurotoxic effects through a few key mechanisms that impair cognitive function in *α*‐synucleinopathies.^[^ ^4^ ^]^ Of these mechanisms, the one that has been demonstrated most clearly is the activation of NMDA receptors via the formation of a complex between PrPC and *α*‐Syn oligomers, resulting in the induction of synaptic impairment and cognitive disorders;^[^ ^4d^ ^]^ however, *α*‐Syn fibrils do not possess this neurotoxicity.^[^ ^4d^ ^]^ Consequently, it remains unclear whether *α*‐Syn fibrils impair cognitive function through other mechanisms in *α*‐synucleinopathies. Some clinical studies have provided key evidence for the role of vascular pathologies in the cognitive defects seen in *α*‐synucleinopathies.^[^ ^5^ ^]^ For example, a multicenter prospective observational longitudinal cohort study reported in *Movement Disorders* highlighted the association between vascular risk factors and PD cognitive impairment.^[^ ^5a^ ^]^ In addition, existing evidence demonstrates that increased plasma *α*‐Syn levels are associated with cognitive impairment in *α*‐synucleinopathies, including PD, MSA, and DLB.^[^ ^6^ ^]^ Nevertheless, why higher plasma *α*‐Syn levels influence cognition and how *α*‐Syn transmission affects cognition in *α*‐synucleinopathies are still not fully understood. Some studies have provided evidences for the transmission of peripheral blood *α*‐Syn into the brain, as exogenous *α*‐Syn fibrils injected intravenously can be detected in the brain after a period of time.^[^ ^7^ ^]^ Brain microvascular endothelial cells (BMVECs) are the cells in the cerebral microvessels that have direct physical contact with the peripheral blood; these cells may act as a bridge between plasma *α*‐Syn and pathological brain *α*‐Syn in *α*‐synucleinopathies. It has been demonstrated that *α*‐Syn fibrils can form a nucleation point for *α*‐Syn inclusions and induce the spread of these inclusions in neurons, and they have been shown to form the specific initiating structure of the Lewy body and to lead to apoptosis or mediator‐dependent neuronal death in vitro and in vivo.^[^ ^8^ ^]^ We wondered whether *α*‐Syn fibrils induce the spread of inclusions and the initiation of mediator‐dependent cell death in BMVECs. Overall, it is worthwhile to explore the connection among *α*‐Syn‐related pathological processes, vascular pathology caused by injury of BMVECs, and cognitive impairment.

Previously, a study by our team^[^ ^9^ ^]^ has indicated that brain capillaries are damaged in an *α*‐Syn preformed fibril (PFF)‑induced PD model with cognitive impairment, which attracted our attention to the relationship between vascular pathology caused by injury of BMVECs and cognitive impairment in an *α*‐Syn PFF‑induced *α*‐synucleinopathy model. Accordingly, we hypothesized that *α*‐Syn fibrils might influence cognitive function via microvascular effects.

However, our previous study failed to provide specific evidence that *α*‐Syn PFF‑induced cerebral cerebrovascular injury is the cause of cognitive impairment in that model and especially whether it is independent of neuronal damage, as neuronal damage is also induced by *α*‐Syn PFFs.^[^ ^9^ ^]^ To overcome this limitation, two models, consisting of adeno‐associated virus (AAV)‐*α*‐Syn and *α*‐Syn PFF injections, are now included so that the effects of nerve‐mediated and microvasculature‐mediated injury can be considered independently. Based on previous studies using *α*‐synucleinopathy models,^[^ ^10^ ^]^ we constructed various *α*‐synucleinopathy pathology models to observe their effects on cognitive function. We found that injection of AAV‐*α*‐Syn or *α*‐Syn PFFs into the unilateral substantia nigra (SN), hippocampus, and cerebral cortex could cause significantly impaired spatial learning and memory retention at 6 months post‐injection.

At 6 months post‐injection, the loss of neurons in mice injected with *α*‐Syn PFFs was relatively mild compared with that in mice injected with AAV‐*α*‐Syn, but the impairments of the cerebral microvascular system (CMS), neurovascular unit (NVU) coupling, and the blood–brain barrier (BBB) were more serious. This result indicated that the cerebral microvascular injury caused by *α*‐Syn PFFs might be relatively independent of neuronal damage in this mouse model of *α*‐synucleinopathies. We further investigated the underlying correlation between vascular injury and *α*‐Syn fibrils. *α*‐Syn PFFs were found to enter primary cultured mouse BMVECs through lymphocyte‐activation gene 3 (Lag3)‐dependent endocytosis in vitro, causing endothelial dysfunction and poly(ADP‐ribose) (PAR)‐driven cell death. Furthermore, the cognitive impairment and cerebral microvascular injury caused by *α*‐Syn PFFs were reversed by deletion of endothelial cell‐specific Lag3 in vivo; therefore, our study provides the key evidence for the inference that cerebral microvascular injury induced by *α*‐Syn fibrils independently exacerbates cognitive impairment in *α*‐synucleinopathies.

## 2 Results

### 2.1 Compared with AAV‐Induced Pathology, *α*‐Syn PFFs‐Induced Pathology in C57BL/6 Mice Damages Cognitive Function with Mild Neuronal Damage

Two mouse models, namely, mice injected with an adeno‐associated virus vector (AAV/2) overexpressing the human A53T *α*‐Syn gene (AAV‐A53T) and mice injected with mouse *α*‐Syn PFFs in the left SN, hippocampus, and cerebral cortex (the specific injected brain regions are marked in Figure S1A, Supporting Information), were generated to replicate *α*‐Syn pathology. For both *α*‐Syn groups, impairment of spatial learning and memory abilities appeared at 6 months post‐injection. At 6 months post‐injection, there was an obvious difference in neuron damage and microvascular injury in the two *α*‐Syn pathology groups, enabling the independent consideration of the effects of nerve and microvascular‐mediated injury. Thus, 6 months post‐injection was set as the time point to study cognitive function.

**Figure 1:**
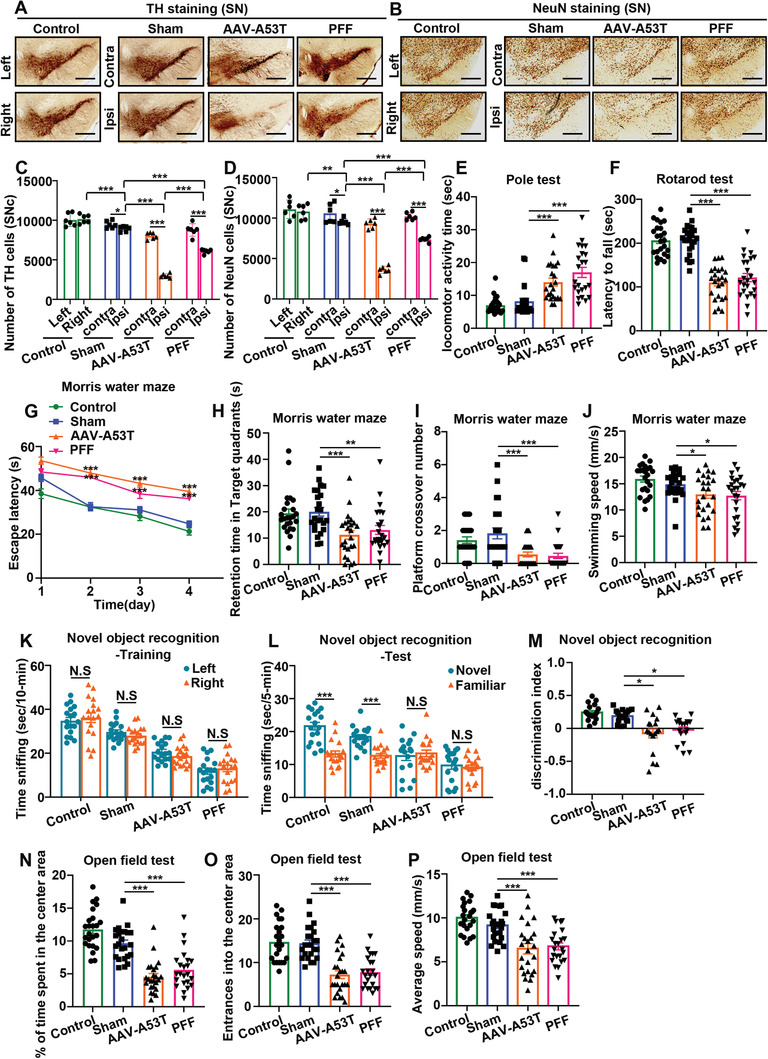
Compared with AAV, *α*‐Syn PFFs‐induced pathology in C57BL/6 mice damages cognitive function with mild neuronal damage. Mice were grouped as follows: control mice (control), negative control AAV/2‐injected mice (sham), AAV/2‐A53T‐injected mice (AAV‐A53T), and *α*‐Syn PFFs injected mice (PFF). A,B) Representative immunohistochemical staining in the SN for A) TH and B) NeuN. The scale bar denotes 500 µm. C,D) Numbers of C) TH cells and D) NeuN cells in the SNc determined by unbiased stereological counting. *p < 0.05, **p < 0.01, ***p < 0.001 (*n* = 6). E) Pole test: scatter plots showing the locomotor activity time (s). ***p < 0.001 (*n* = 24). F) Rotarod test: scatter plots showing the latency to fall (s). ***p < 0.001 (*n* = 24). G–J) MWM maze test: G) escape latency (s), ***p < 0.001 for the AAV‐A53T group or PFF group versus sham group from day 2 onward (*n* = 24); H) scatter plots showing the retention time in the target quadrant on day 5, **p < 0.01, ***p < 0.001 (*n* = 24); I) scatter plots showing the number of platform crossings on day 5, ***p < 0.001 (*n* = 24); J) scatter plots showing the swimming speed on day 5, *p < 0.05 (*n* = 24). K–M) NOR test: K) scatter plots showing the time spent sniffing the left or right object out of 10 min during the training period, N.S. means no significance (*n* = 18); L) scatter plots showing the time spent sniffing the novel or familiar object out of 5 min during the test period, N.S. means no significance, ***p < 0.001 (*n* = 18); M) scatter plots showing the discrimination index in the test period, *p < 0.05 (*n* = 18). N–P) Open field test: N) scatter plots showing the percentage of total time spent in the central area, ***p < 0.001 (*n* = 24); O) scatter plots showing the number of entries into the central area, ***p < 0.001 (*n* = 24); P) scatter plots showing the average speed (cm s^−1^) of mice, ***p < 0.001 (*n* = 24). All data are expressed as the mean ± SEM. Differences among multiple means, one‐way analysis of variance (ANOVA) followed by Bonferroni's post hoc test; differences between 2 means, two‐tailed Student's *t* tests followed by Tukey's post hoc test.

The numbers of tyrosine hydroxylase (TH)^+^ and NeuN^+^ neurons, reflecting the impact of neuronal damage, were consistent with the results from previous studies.^[^ ^11^ ^]^ At 6 months post‐injection, *α*‐Syn PFFs triggered relatively mild neuronal damage, while AAV‐A53T caused more severe neuronal damage (**Figure** **1**A–D). To accurately determine the time point of neuronal injury in those two mouse models, the TH^+^ DA neurons (DAns) were quantified at 1, 2, 3, 4, 5, and 6 months after modeling. The number of TH^+^ DAns in the AAV‐A53T group was reduced by 64.33% at the 3‐month time point, while the tendency of TH^+^ DAn loss was relatively moderate at 4–6 months, with a reduction of 72.1% at 6 months. The number of TH^+^ DAns in the PFF group was decreased by 24.66% at the 3‐month time point and continued to decrease by 4–6 months, with a 41.84% decrease at 6 months (Figure S1E,F,H, Supporting Information).

The rotarod and pole tests were used to evaluate motor deficits. Compared with those in the sham group, the mice in the AAV‐A53T and PFF groups exhibited motor deficits (Figure 1E,F).

The Morris water maze (MWM) and novel object recognition (NOR) test were applied to assess cognitive impairment. In the MWM test, the mice in the AAV‐A53T and PFF groups needed more time to find the platform on days 2–4, spent less time in the target quadrant, performed fewer platform crossovers, and had a slower swimming speed on day 5 than the mice in the sham group (Figure 1G–J). In the training period of the NOR test, mice in all groups lacked any apparent side preference; in the test period of the NOR procedure, the mice in the control and sham groups exhibited a preference for the novel object, whereas the mice in the AAV‐A53T and PFF groups did not exhibit that preference (Figure 1K,L). In addition, the discrimination index values of the AAV‐A53T and PFF groups in the NOR test were lower than that of the sham group (Figure 1M). The above results indicated that spatial learning and memory retention were hindered in the *α*‐Syn pathology groups compared with the sham group and that *α*‐Syn PFF injection severely impaired spatial learning and memory retention.

The open field test was performed to reflect emotional disorders. The results revealed that the mice in the AAV‐A53T and PFF groups spent more time on the edge of the open field and made fewer entries into the center of the open field than the mice in the sham group (Figure 1N,O). The average movement speed of the AAV‐A53T and PFF groups was slower than that of the sham group (Figure 1P). This result suggested that *α*‐Syn pathology in mice, especially that induced by *α*‐Syn PFF injection, caused disorder of motor and emotion‐related neural circuits. Representative track maps of the MWM, NOR, and open field test are shown in Figure S1B–D in the Supporting Information.

### 2.2 Compared with AAV‐Induced Pathology, *α*‐Syn PFF‐Induced Pathology in C57BL/6 Mice Caused More Severe CMS Damage, Less Glial Inflammation, and Fewer *α*‐Syn Aggregates (Oligomers)

The capillary vessel network, cerebral microvascular density, volume/diameter of classified vessels (vein and artery), and percentage of microvascular pericyte coverage were evaluated to reflect changes in the CMS.

First, to reveal cerebrovascular injury at different time points after AAV‐A53T or PFF modeling, capillary vessel imaging via an in vivo injection of fluorescein isothiocyanate (FITC)‐dextran exhibiting the vascular network density was performed at 1, 2, 3, 4, 5, and 6 months after injection. Specifically, the microvascular perfusion in the AAV‐A53T group showed 20.94% and 35.49% reductions at the 3‐ and 6‐month time points, respectively, and that in the PFF group showed 53.42% and 75.32% reductions, respectively (Figure S1G,I, Supporting Information). When these data were combined with the data on neuron counts, cerebrovascular injury and neuronal injury were found to be relatively independent.

**Figure 2:**
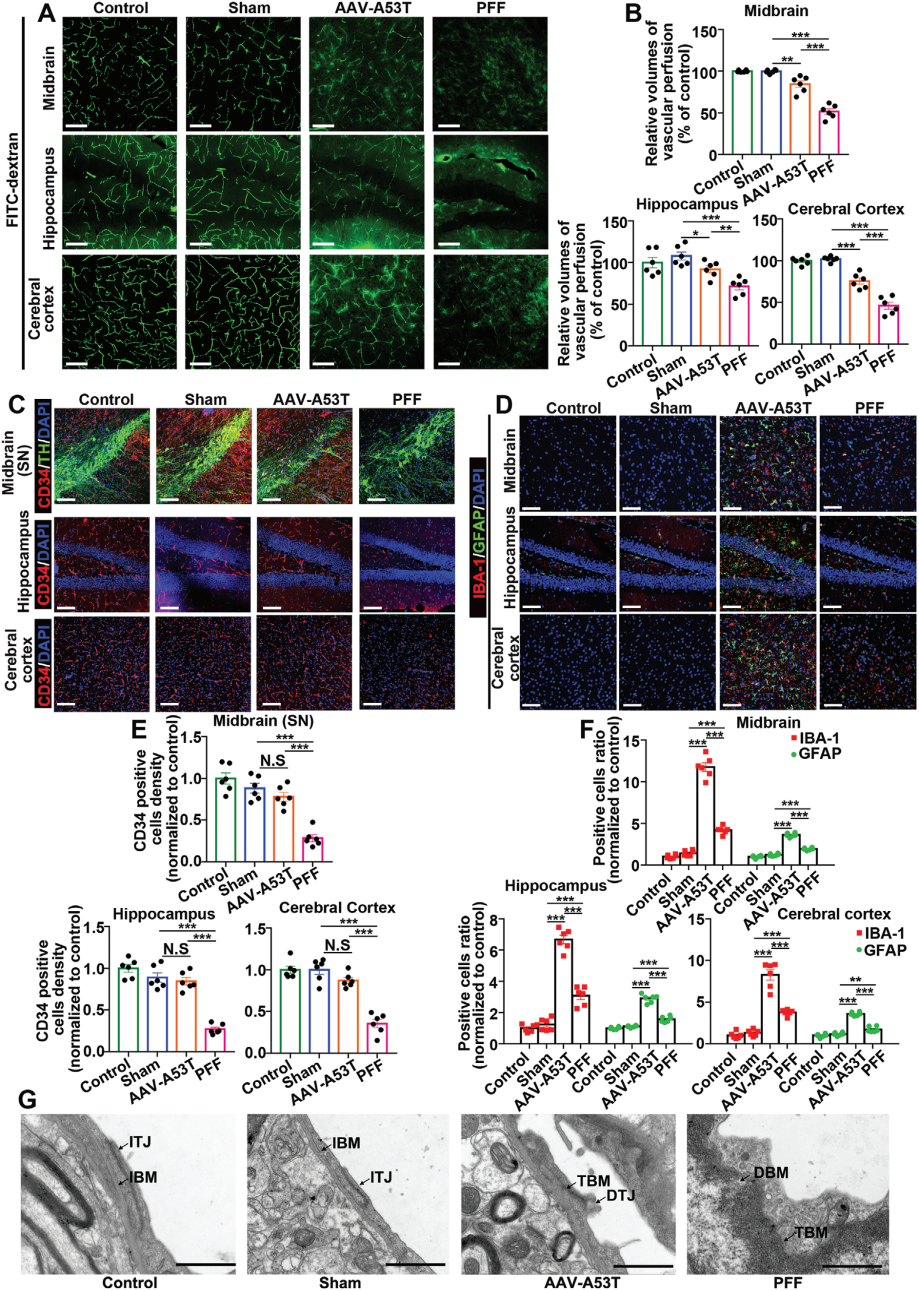
*α*‐Syn PFF‐induced pathology in C57BL/6 mice caused more severe CMS damage, less glial inflammation, and fewer *α*‐Syn aggregates (oligomers) than AAV expression. A) Representative vessel imaging of FITC‐dextran perfusion in the midbrain, hippocampus, and cerebral cortex for each group. The scale bar denotes 100 µm. B) Scatter plots showing the relative volumes of FITC‐dextran‐marked perfusion (% of control group) in the midbrain, hippocampus, and cerebral cortex for each group. ***p < 0.001. The data are expressed as the mean ± SEM. One‐way ANOVA with Bonferroni's post hoc test (*n* = 6). C) Representative immunofluorescence costaining images of CD34 and TH in the midbrain (SN) and representative immunofluorescence staining images of CD34 in the hippocampus and cerebral cortex for each group. The scale bar denotes 100 µm. D) Representative immunofluorescence costaining images of IBA1 and GFAP in the midbrain, hippocampus, and cerebral cortex for each group. The scale bar denotes 100 µm. E) Quantification of (C). The scatter plots show the CD34‐positive cell density (normalized to control) in the midbrain, hippocampus, and cerebral cortex for each group. N.S. means no significance, ***p < 0.001. The data are expressed as the mean ± SEM. One‐way ANOVA with Bonferroni's post hoc test (*n* = 6). F) Quantification of (D). The scatter plots show the positive cell ratio of IBA1 or GFAP (normalized to control) in the midbrain, hippocampus, and cerebral cortex for each group. ***p < 0.001. The data are expressed as the mean ± SEM. One‐way ANOVA with Bonferroni's post hoc test (*n* = 6). G) Representative TEM imaging of endothelial basement membranes of each group. IBM: intact basement membrane; TBM: thickened basement membrane; DBM: detached basement membrane; ITJ: intact tight junction; DTJ: discontinuous tight junction. The scale bar denotes 500 nm.

In the following study, the data 6 months after injection were analyzed in detail. Capillary vessel imaging of different brain regions (the midbrain, hippocampus, and cerebral cortex) was performed (**Figure** **2**A,B). The vascular perfusion volumes of the *α*‐Syn pathology groups were obviously decreased compared to those in the sham group. Furthermore, the vascular perfusion volume of the PFF group was distinctly lower than that of the AAV‐A53T group. The CD34 immunopositive cell density was detected to reflect the cerebral microvascular density.^[^ ^12^ ^]^ The decrease in CD34‐positive cell density in the PFF group was stronger than that in the sham and AAV‐A53T groups (Figure 2C,E). In addition, costaining of CD31 and *α*‐smooth muscle actin (*α*‐SMA) was performed to identify classified vessels (veins and arteries).^[^ ^13^ ^]^ The volume and diameter of vein/artery vessels in the PFF group were significantly smaller than those in the sham and AAV‐A53T groups (Figure S2A,C–H, Supporting Information). Damage to capillaries alters pericytes, and the percentage of microvascular pericyte coverage was measured by costaining for neural/glial antigen 2 (NG2, a marker for pericytes) and CD31.^[^ ^14^ ^]^ The microvascular pericyte coverage percentage of the *α*‐Syn pathology groups was obviously less than that of the sham group, and the microvascular pericyte coverage percentage of the PFF group was less than that of the AAV‐A53T group (Figure S2B,I–K, Supporting Information). The above indictors for the CMS indicated that *α*‐Syn PFFs led to more severe microvascular CMS injury than AAV‐A53T.

To study whether CMS injury is accompanied by glial inflammation, glial activation was measured by costaining of ionized calcium binding adapter molecule 1 (IBA1) and glial fibrillary acidic protein (GFAP).^[^ ^15^ ^]^ As shown in Figure 2D,F, the IBA1/GFAP‐positive cell ratio of the *α*‐Syn pathology groups was increased, but surprisingly, although there was more severe CMS injury in the PFF group, the increase in the IBA1/GFAP‐positive cell ratio in the PFF group was much smaller than that in the AAV‐A53T group, indicating that *α*‐Syn PFFs led to more mild glial inflammation than AAV‐A53T. Since neurotoxic *α*‐Syn aggregates (oligomers) are responsible for exacerbated neuron loss and glial activation,^[^ ^16^ ^]^ it is necessary to classify the different *α*‐Syn aggregate species in the two *α*‐Syn pathology groups. *α*‐Syn aggregates were detected using homogenous time‐resolved fluorescence (HTRF)^[^ ^17^ ^]^ in the three brain regions of interest. As shown in Figure S2L–N in the Supporting Information, significantly higher fluorescence resonance energy transfer (FRET) signals were detected in three brain regions from the AAV‐A53T group than those from the PFF group, indicating that AAV‐A53T infection produced more *α*‐Syn aggregates (oligomers) than *α*‐Syn PFFs.

Capillary ultrastructure was analyzed using transmission electron microscopy (TEM), as previously described.^[^ ^18^ ^]^ Representative TEM images are shown in Figure 2G, the quantified values are shown in Table S1 in the Supporting Information. Capillary ultrastructure damage was consistently more severe in the PFF group than in the sham and AAV‐A53T groups.

### 2.3 Compared with AAV‐Induced Pathology, *α*‐Syn PFFs‐Induced Pathology in C57BL/6 Mice Induced More Critical Impairment of NVU Coupling

NVU coupling has been reported to reflect the functioning of the CMS.^[^ ^19^ ^]^ It is mediated by contact between astrocytic end‐feet (measured by AQP4) and cerebral blood vessels (measured by collagen IV, Collv).^[^ ^20^ ^]^ The integrity of the contact between cerebral blood vessels and astrocytic end‐feet was assessed by colocalization of AQP4 and collagen IV to evaluate NVU coupling.^[^ ^21^ ^]^

**Figure 3:**
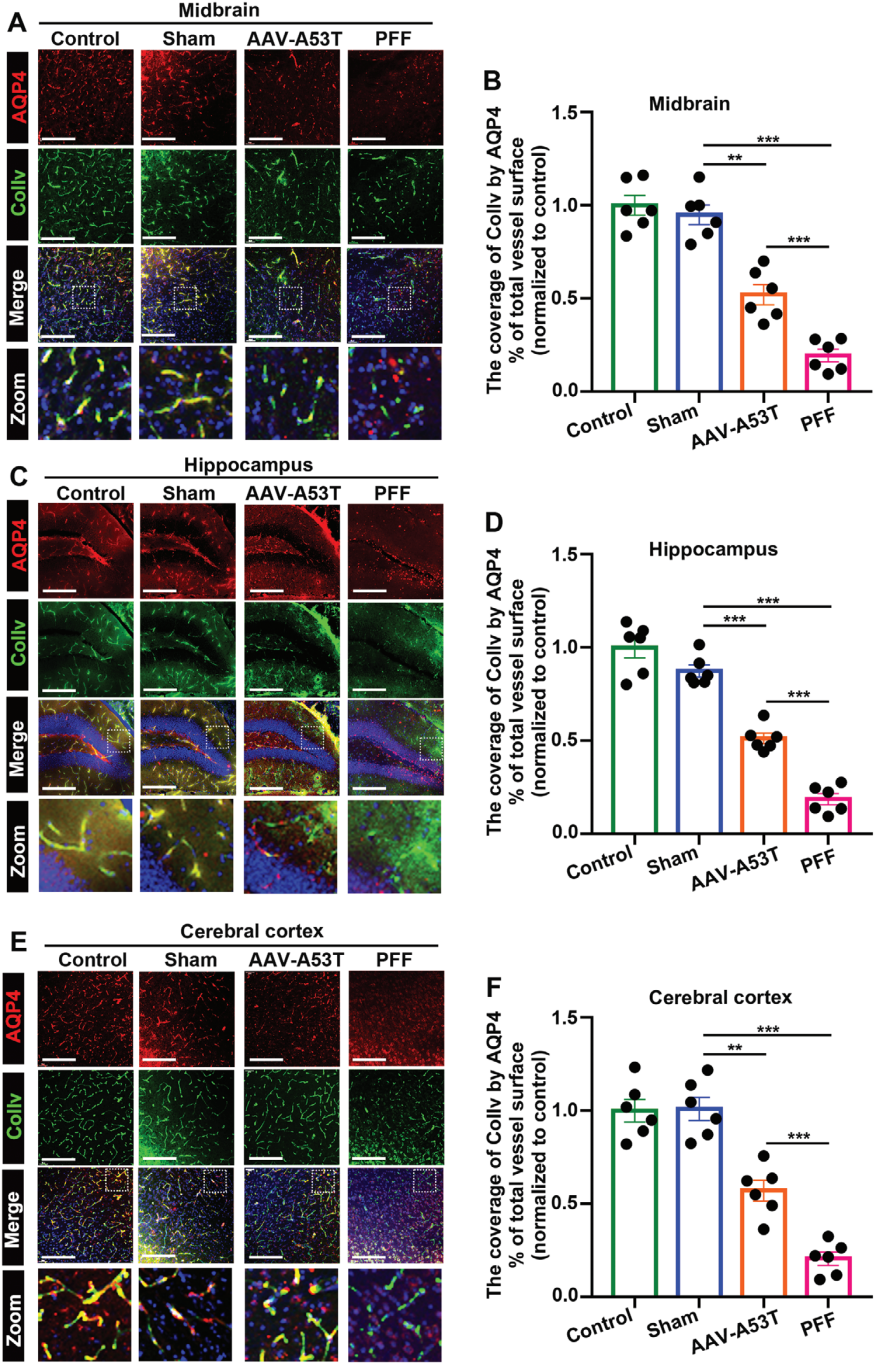
*α*‐Syn PFF‐induced pathology in C57BL/6 mice induced more critical impairment of NVU coupling than AAV expression. A,C,E) Representative immunofluorescence costaining images of AQP4 and collagen IV and a merged image (AQP4/Collv/DAPI) in the A) midbrain, C) hippocampus, and E) cerebral cortex for each group. The scale bar denotes 200 µm. B,D,F) Quantification of (A, C, E). The scatter plots show the coverage of cortical blood vessels (Collv) by astrocytic end‐feet AQP4 (% of total vessel surface normalized to control group) in the B) midbrain, D) hippocampus, and F) cerebral cortex for each group. **p < 0.01, ***p < 0.001. The data are expressed as the mean ± SEM. One‐way ANOVA with Bonferroni's post hoc test (*n* = 6).

Typical confocal maps in the midbrain, hippocampus, and cerebral cortex of each group (**Figure** **3**A,C,E) and the statistical results of vascular AQP4 staining (% of total vessel surfaces with staining normalized to that of the control group) (Figure 3B,D,F) are shown to illustrate the observed effect. Compared with the sham group, the AAV‐A53T and PFF groups displayed distinctly lower AQP4 levels in blood vessels. Furthermore, there was distinctly less colocalization of AQP4 and collagen IV in the PFF group than in the AAV‐A53T group, revealing the significant effects of *α*‐Syn PFF‐induced pathology on NVU coupling.

### 2.4 Compared with AAV Injection, Injection of *α*‐Syn PFFs Caused a More Significant Decrease in Tight Junction Protein Expression and a More Significant Increase in BBB Permeability in C57BL/6 Mice

In the nervous system, microvascular endotheliocytes are central components of the NVU that regulate the BBB to maintain functional homeostasis of the brain.^[^ ^19^ ^]^ Evidence suggests that BBB breakdown is an early biomarker of human cognitive dysfunction.^[^ ^22^ ^]^ Therefore, we next investigated whether the BBB was damaged in our models.

**Figure 4:**
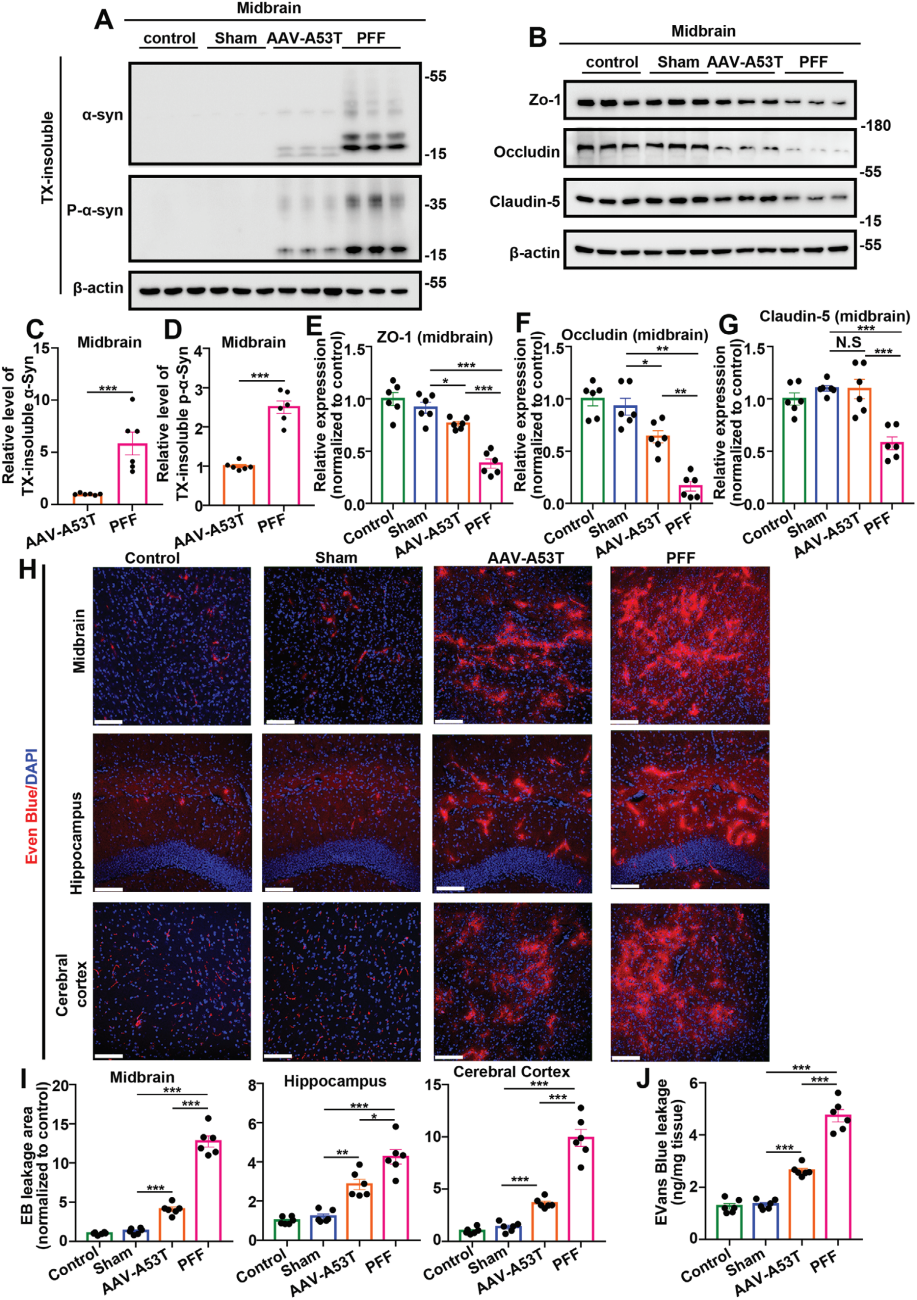
Injection of *α*‐Syn PFFs caused a more significant decrease in tight junction protein expression and increase in BBB permeability than AAV expression in C57BL/6 mice. A) Representative western blot images of TX‐insoluble *α*‐Syn and P‐*α*‐Syn. Protein lysates derived from midbrain tissues of each group were extracted in 1% TX‐100 (TX‐soluble) followed by 2% SDS (TX‐insoluble). B) Representative western blot imaging of tight junction proteins in midbrain tissues. C,D) Quantification of (A). The scatter plots show the relative levels of TX‐insoluble C) *α*‐Syn and D) P‐*α*‐Syn at 6 months after injection of AAV‐A53T or *α*‐Syn PFFs. ***p < 0.001. The data are expressed as the mean ± SEM. Two‐tailed Student's *t* tests followed by Tukey's post hoc test (*n* = 6). E–G) Quantification of (B). The scatter plots show the relative expression of tight junction proteins in midbrain tissues (normalized to control) of each group, including E) Zo‐1, F) Occludin, and G) Claudin‐5. N.S. means no significance, *p < 0.05, ***p < 0.01, ***p < 0.001. The data are expressed as the mean ± SEM. One‐way ANOVA with Bonferroni's post hoc test (*n* = 6). H) Representative EB extravasation imaging (Evans Blue/DAPI) in the midbrain, hippocampus, and cerebral cortex of each group. The scale bar denotes 100 µm. I) Quantification of (H). The scatter plots show the relative EB leakage area (normalized to control) of each group. *p < 0.05, ***p < 0.001. The data are expressed as the mean ± SEM. One‐way ANOVA with Bonferroni's post hoc test (*n* = 6). J) Scatter plots show the relative EB leakage measured by EB content of the whole injected hemisphere of each group. ***p < 0.001. The data are expressed as the mean ± SEM. One‐way ANOVA with Bonferroni's post hoc test (*n* = 6).

First, the pathological insoluble *α*‐Syn accumulation provoked by AAV‐A53T and PFF injection was observed as a reflection of pathological *α*‐Syn in each group, which might be relevant to BBB impairment.^[^ ^23^ ^]^ The expression of insoluble *α*‐Syn and phospho‐*α*‐Syn (P‐*α*‐Syn) in the midbrain was induced by AAV‐A53T and PFF injection. In addition, the degree of insoluble *α*‐Syn/P‐*α*‐Syn accumulation was greater in the PFF group than in the AAV‐A53T group (**Figure** **4**A,C,D).

To assess the BBB, we analyzed tight junction protein expression, and then tested in vivo BBB permeability via intravenous tail vein injection of Evans Blue (EB) and two sizes of fluorescence‐labeled dextran (FITC, 40 kDa as an intermediate size and 70 kDa as a large size).

To analyze tight junction protein expression, the levels of ZO‐1, Occludin, and Claudin‐5 were measured by western blot analysis, and the levels of the corresponding mRNAs were measured by quantitative real‐time polymerase chain reaction (qRT‐PCR). The data from the western blot analysis are shown in Figure 4B,E,F,G and Figure S3A–J in the Supporting Information. As shown, in the PFF group, the expression of all tight junction proteins was obviously reduced, while in the AAV‐A53T group, only the expression of Occludin in the midbrain, hippocampus, and cerebral cortex and the expression of Zo‐1 in the midbrain were decreased. In addition, the relative expression of all tight junction proteins in the PFF group was lower than that in the AAV‐A53T group. The mRNA expression of tight junction proteins measured by qRT‐PCR revealed the same trend as protein expression (Figure S3K–M, Supporting Information). These results indicate that *α*‐Syn PFFs can lead to more severe tight junction protein impairment than AAV‐A53T.

To analyze in vivo BBB permeability, EB fluorescence (red) and 4′,6‐diamidino‐2‐phenylindole (DAPI) costaining (to observe the EB leakage area) and quantification of EB leakage in the whole hemisphere were performed.^[^ ^24^ ^]^ As shown in Figure 4H–J, the EB leakage in the two *α*‐Syn pathology groups was visibly higher than that in the sham group. In addition, the EB leakage in the PFF group was higher than that in the AAV‐A53T group. Furthermore, two fluorescence‐labeled dextrans with a larger molecular weight (40 and 70 kDa) than EB (0.96 kDa) were applied to further detect BBB permeability. The two different sizes of fluorescence‐labeled dextran also passed through the damaged BBB (Figure S4A–G, Supporting Information), and there was no significant difference in fluorescence leak in the brain parenchyma of the two sizes (Figure S4H–J, Supporting Information). These results indicate that *α*‐Syn PFFs triggered a larger increase in BBB permeability than AAV‐A53T.

### 2.5 Injected Exogenous *α*‐Syn PFFs Could be Taken Up by Endothelial Cells In Vivo

To further investigate and provide insights into the mechanisms by which *α*‐Syn PFFs cause vascular injury, we tested whether exogenous *α*‐Syn PFFs could be taken up into endothelial cells (ECs). The *α*‐Syn‐biotin PFF was used to identify exogenously injected *α*‐Syn PFFs in the mouse brain.

**Figure 5:**
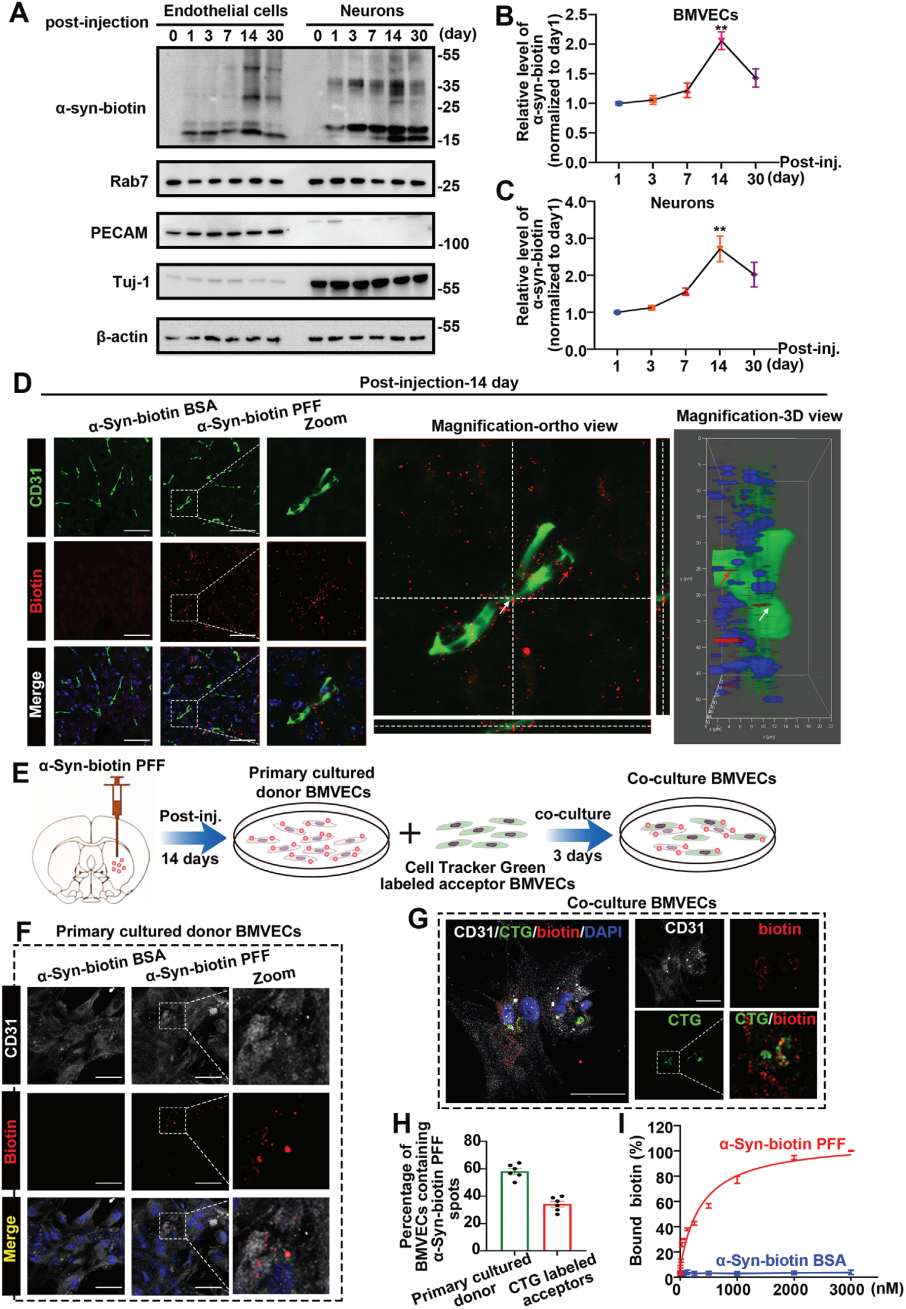
ECs took up exogenous *α*‐Syn PFFs in vivo. A) Representative western blot imaging of *α*‐Syn‐biotin PFFs in the endosome‐enriched proteins of neuronal tissue or brain microvascular tissue at each time point (the WT mice injected with *α*‐Syn‐biotin PFFs were sacrificed 1, 3, 7, 14, and 30 days later). Rab7 was used to confirm the isolation of endosomes. PECAM and TUJ‐1 antibodies were used in combination to detect the purity of isolated brain microvascular tissue (PECAM, specifically expressed in BMVECs) or neuronal tissue (TUJ‐1, specifically expressed in neurons). B,C) Quantification of (A). The line graph shows the relative level of *α*‐Syn‐biotin PFF normalized to day 1 in B) ECs or C) neurons. Compared to the level as of day 1, the relative level of *α*‐Syn‐biotin PFF was the highest on day 14. **p < 0.01. The data are expressed as the mean ± SEM. Two‐tailed Student's *t* tests followed by Tukey's post hoc test (three independent replicates). D) Representative immunofluorescence costaining images of biotin and CD31 in WT mice 14 days after *α*‐Syn‐biotin PFF injection (*α*‐Syn‐biotin BSA was used as a negative control). Magnified ortho view and 3D view of zoom are displayed. The arrows indicate the internalized *α*‐Syn‐biotin PFF. The scale bar denotes 50 µm. E) Schematic diagram of the experimental design of *α*‐Syn‐biotin PFF internalization and transfer between BMVECs. Primary cultured donor BMVECs extracted from WT mice 14 days after injection with *α*‐Syn‐biotin PFFs were thoroughly washed, detached, and cocultured with CellTracker Green (CTG)‐labeled acceptor BMVECs (labeled in suspension) for 72 h. Then, the cells were fixed, stained, and imaged. F) Representative immunofluorescence costaining images of CD31 and biotin for primary cultured donor BMVECs extracted from WT mice 14 days after injection with *α*‐Syn‐biotin PFFs (*α*‐Syn‐biotin BSA was used as a negative control). The scale bar denotes 50 µm. G) Representative confocal image showing the donor and acceptor BMVECs in coculture after 72 h. The donor BMVECs were primary cultured BMVECs extracted from WT mice 14 days after injection with *α*‐Syn‐biotin PFFs, and the acceptor BMVECs were BMVECs labeled with CTG. The cells were labeled with anti‐CD31, and the nuclei were counterstained with DAPI. The smaller panels on the right show images of the same field in different channels: CD31 (white), biotin (red), CTG (green), and CTG/biotin. Intracellular localization of *α*‐Syn‐biotin PFF in acceptor BMVECs was confirmed with the localization of the *α*‐Syn‐biotin PFF and CTG in the channel images of CTG/Biotin. The scale bar denotes 50 µm. H) Scatter plots show the percentage of primary cultured donor BMVECs or CTG‐labeled acceptor BMVECs containing *α*‐Syn‐biotin PFF puncta. The data show the mean ± SEM from six independent experiments. I) Dissociation curve of *α*‐Syn‐biotin PFF or *α*‐Syn‐biotin BSA binding to primary BMVECs. The concentrations of *α*‐Syn‐biotin PFF were set up with *α*‐Syn‐biotin monomer equivalents (× 10^−9^ m) in 0.1% TX‐100. The data are expressed as the mean ± SEM (three independent replicates).

The mice injected with *α*‐Syn‐biotin PFF were sacrificed 1, 3, 7, 14, and 30 days later, and western blot analysis was performed to detect the expression of *α*‐Syn‐biotin in the endosome‐enriched proteins of neuronal tissue or brain microvascular tissue at each time point. Accumulated *α*‐Syn‐biotin was detectable in both neurons and ECs, and the maximum expression of exogenous *α*‐Syn‐biotin occurred 14 days after injection (**Figure** **5**A–C). Furthermore, exogenous biotin in CD31^+^ ECs could be tracked 14 days after *α*‐Syn‐biotin PFF injection (*α*‐Syn‐biotin BSA (bovine serum albumin) was used as a negative control) (Figure 5D).

To observe the transfer efficiency of the exogenous PFF between BMVECs more accurately, as previously described,^[^ ^25^ ^]^ the BMVECs derived from the mice with *α*‐Syn‐biotin PFF injected for 14 days (as donor BMVECs) were cocultured with Cell Tracker Green‐labeled acceptor BMVECs, and the percentages of BMVECs containing *α*‐Syn‐biotin spots in donor or acceptor BMVECs were quantified as indicators of the transfer efficiency between BMVECs (Figure 5E–H). The data showed that the exogenous PFF could be transmitted between BMVECs. Then, the dissociation curve of *α*‐Syn‐biotin PFF binding to primary BMVECs was assessed (Figure 5I and Figure S5A, Supporting Information). The above data provide evidence that ECs are able to take up exogenous *α*‐Syn.

### 2.6 Introduction of Exogenous *α*‐Syn PFFs Initiated the Formation of Insoluble *α*‐Syn Inclusions via Lag3‐Dependent Endocytosis in Primary BMVECs In Vitro

**Figure 6:**
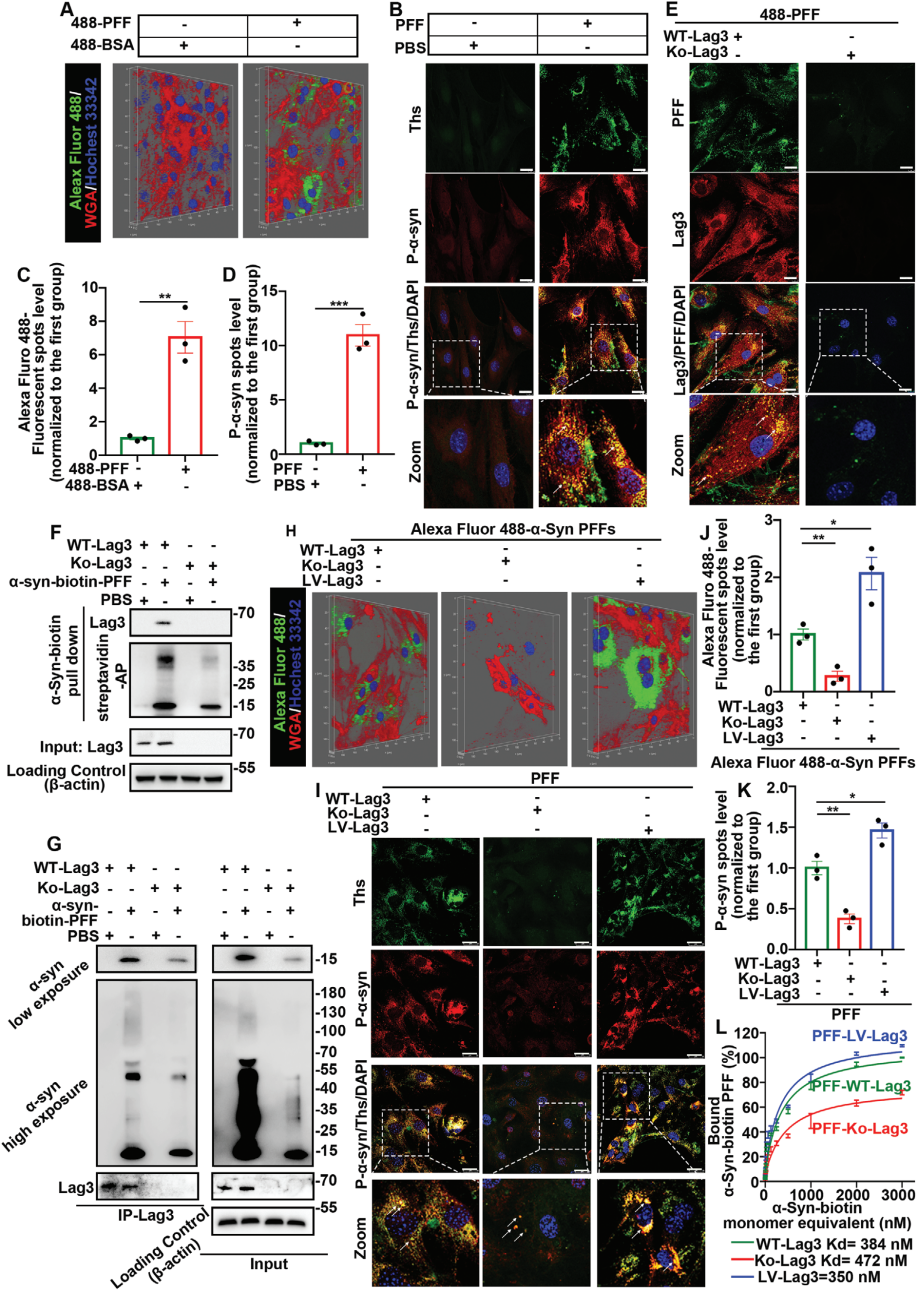
Exogenous *α*‐Syn PFFs contributed to insoluble *α*‐Syn inclusions via Lag3‐dependent endocytosis. A) Representative confocal 3D imaging of live cell staining to capture images of exogenous *α*‐Syn PFFs entering BMVECs. The cells were treated with 488‐PFF or 488‐BSA for 48 h. Alexa Fluor 488‐tagged *α*‐Syn PFFs were used to track exogenous *α*‐Syn PFFs (Alexa Fluor 488‐tagged BSA was used as a negative control), Alexa Fluor 555‐wheat germ agglutinin (WGA) dye was used as a cytoskeleton marker, and Hoechst 33342 was used to stain nuclei. Cells were washed with PBS three times to remove the free biotin. B) Representative costaining images of P‐*α*‐Syn and ThS in BMVECs treated with 488‐PFFs or 488‐BSA for 14 days. The colocalization of *α*‐Syn and ThS was clearly observed, as indicated by the white arrows. The scale bar denotes 20 µm. C) Quantification of (A). The scatter plots show the relative Alexa Fluor 488 fluorescent spot levels (normalized to the first group). **p < 0.01. The data are expressed as the mean ± SEM. Two‐tailed Student's *t* tests followed by Tukey's post hoc test (three independent replicates). D) Quantification of (B). The scatter plots show the relative P‐*α*‐Syn level (normalized to the first group). ***p < 0.001. The data are expressed as the mean ± SEM. Two‐tailed Student's *t* tests followed by Tukey's post hoc test (three independent replicates). E) Representative images of Alexa Fluor 488‐tagged PFFs and Lag3 in BMVECs derived from WT mice or Lag3^−/−^ mice treated with Alexa Fluor 488‐*α*‐Syn PFFs for 48 h. The colocalization of Alexa Fluor 488‐tagged PFFs and Lag3 was clearly observed, as indicated by the white arrows, in BMVECs derived from WT mice but hardly observed in BMVECs derived from Lag3^−/−^ mice. The scale bar denotes 20 µm. F,G) Co‐IP analysis of products from BMVECs extracted from WT or Lag3^−/−^ mice treated with *α*‐Syn‐biotin PFFs for 1 week (PBS was used as a negative control). *β*‐Actin was used as the loading control. F) *α*‐Syn‐biotin PFFs bind to Lag3 as determined by streptavidin beads that pull down *α*‐Syn‐biotin PFFs. G) LAG3 binds *α*‐Syn‐biotin PFF as detected by anti‐LAG3 antibody. *α*‐Syn expression in the Co‐IP products is displayed under both low and high exposure. H–L) Cells were primary BMVECs extracted from WT or Lag3^−/−^ mice or LV‐Lag3‐transfected WT mouse BMVECs (cells from Lag3^−/−^ mice and WT mice were transfected with negative control lentivirus). H) Representative confocal 3D imaging of live cell staining of BMVECs. The cells were treated with Alexa Fluor 488‐tagged *α*‐Syn PFFs for 48 h. J) Quantification of (H). The scatter plots show the relative quantities Alexa Fluor 488‐labeled fluorescent puncta (normalized to the first group). *p < 0.05, **p < 0.01. The data are expressed as the mean ± SEM. One‐way ANOVA with Bonferroni's post hoc test (three independent replicates). I) Representative costaining images of P‐*α*‐Syn and ThS in BMVECs treated with PFFs for 48 h. The colocalization of P‐*α*‐Syn and ThS was clearly observed, as indicated by the white arrows. The scale bar denotes 20 µm. K) Quantification of (I). The scatter plots show the relative P‐*α*‐Syn level (normalized to the first group). *p < 0.05, **p < 0.01. The data are expressed as the mean ± SEM. One‐way ANOVA with Bonferroni's post hoc test (three independent replicates). L) The dissociation curve of *α*‐Syn‐biotin PFF binding to primary BMVECs. The concentrations of *α*‐Syn‐biotin PFFs were set up with *α*‐Syn‐biotin monomer equivalents (× 10^−9^ m) in 0.1% TX‐100. The data are expressed as the mean ± SEM (three independent replicates).

Alexa Fluor 488‐tagged *α*‐Syn PFFs were used to track whether exogenous *α*‐Syn PFFs entered BMVECs after introduction for 48 h.^[^ ^2a^ ^]^ 3D laser scanning confocal microscopy of 488‐tagged *α*‐Syn PFFs and 555‐tagged wheat germ agglutinin (WGA) (cytoskeletal marker)^[^ ^26^ ^]^ was conducted to reflect entry (**Figure** **6**A,C). Alexa Fluor 488‐tagged BSA was used as a negative control. The colocalization of Ser129‐phosphorylated *α*‐Syn (P‐*α*‐Syn) and thioflavin S (ThS) was observed as a reflection of insoluble *α*‐Syn inclusions after treatment with *α*‐Syn PFFs for 14 days (Figure 6B,D). The results showed that the introduction of exogenous *α*‐Syn PFFs initiated the formation of insoluble *α*‐Syn after 14 days in primary BMVECs. In addition, exogenous *α*‐Syn PFFs entering primary neurons and insoluble *α*‐Syn inclusions in primary neurons (14 days) were also observed (Figure S6A–D, Supporting Information).

Lag3 has been reported to be a key protein for *α*‐Syn PFF endocytosis in mouse neuron.^[^ ^27^ ^]^ According to the EMBL‐EBI database, Lag3 is expressed in cortical ECs in mice. Immunofluorescence was performed to verify the expression of Lag3 in primary mouse BMVECs (Figure S8E, Supporting Information).

To further verify whether Lag3 in ECs is also the key protein for *α*‐Syn PFF endocytosis, the colocalization of Alexa Fluor 488 and Lag3 was determined in primary BMVECs treated with Alexa Fluor 488‐*α*‐Syn PFFs for 48 h. The colocalization of exogenous PFF and Lag3 was observed in BMVECs derived from wild‐type (WT) mice but hardly observed in BMVECs derived from Lag3^−/−^ mice (Figure 6E). Next, coimmunoprecipitation (Co‐IP) assays were used to determine whether Lag3 directly interacted with the introduced exogenous *α*‐Syn PFFs. Streptavidin‐AP determining *α*‐Syn‐biotin PFF was used to pull down the proteins, and Lag3 was detected in the Co‐IP products (from BMVECs extracted from WT or Lag3^−/−^ mice treated with *α*‐Syn‐biotin PFF for 1 week) (Figure 6F). Moreover, the *α*‐Syn protein was also detectable when Lag3 was pulled down, and *α*‐Syn expression was detected under both low and high exposure conditions, reflecting the depolymerization of macromolecular exogenous *α*‐Syn‐biotin PFF in sodium dodecyl sulfate‐polyacrylamide gels (Figure 6G). Together, these results indicated that Lag3, which interacts with the exogenous *α*‐Syn PFFs directly, is the key protein for the endocytosis of *α*‐Syn PFFs into primary BMVECs.

Next, we modulated Lag3 expression to assess whether it would affect *α*‐Syn PFFs endocytosis and the subsequent formation of insoluble *α*‐Syn inclusions. Primary BMVECs extracted from WT or Lag3^−/−^ mice or LV‐Lag3‐transfected WT mouse BMVECs (cells from Lag3^−/−^ mice and WT mice were transfected with negative control lentivirus) were used. Overexpression of Lag3 increased the endocytosis of Alexa Fluor 488 *α*‐Syn PFFs and the colocalization of P‐*α*‐Syn and ThS, whereas knockout of Lag3 significantly reduced *α*‐Syn PFFs endocytosis and the colocalization of P‐*α*‐Syn and ThS in BMVECs (Figure 6H–K). Furthermore, we designed three shRNAs to knock down the expression of Lag3 in order to determine whether overexpression of Lag3 by lentivirus can enhance the endocytosis of Alexa Fluor 488 *α*‐Syn PFFs after shRNA knockdown. Overexpression of Lag3 consistently reversed the decrease in exogenous PFF endocytosis induced by knockdown of Lag3 (Figure S6E,F, Supporting Information). These results indicate that regulation of Lag3 can affect *α*‐Syn PFF endocytosis and formation of insoluble *α*‐Syn inclusions in BMVECs.

Then, the dissociation curve of the cell surface binding to *α*‐Syn‐biotin PFF regulated by Lag3 was assessed, and the dissociation constant (*K*_d_) was determined (WT‐*K*_d_ = 384 × 10^−9^ m, Ko‐*K*_d_ = 472 × 10^−9^ m, LV‐*K*_d_ = 350 × 10^−9^ m) (Figure 6L). This result indicates that regulation of Lag3 can alter the cell surface binding of *α*‐Syn‐PFFs to BMVECs.

### 2.7 Introduction of Exogenous *α*‐Syn PFFs into BMVECs In Vitro Damaged Epithelial Cell Barrier Permeability and Caused PAR‐Driven Cell Death

To determine the impact of *α*‐Syn PFFs on the epithelial cell barrier integrity and permeability of BMVECs, the protein and mRNA expression of tight junction proteins and transepithelial electrical resistance (TEER) values were tested. In these experiments, primary BMVECs were treated or pretreated with phosphate‐buffered saline (PBS) or 5 µg mL^−1^ *α*‐Syn PFFs for 14 days.

**Figure 7:**
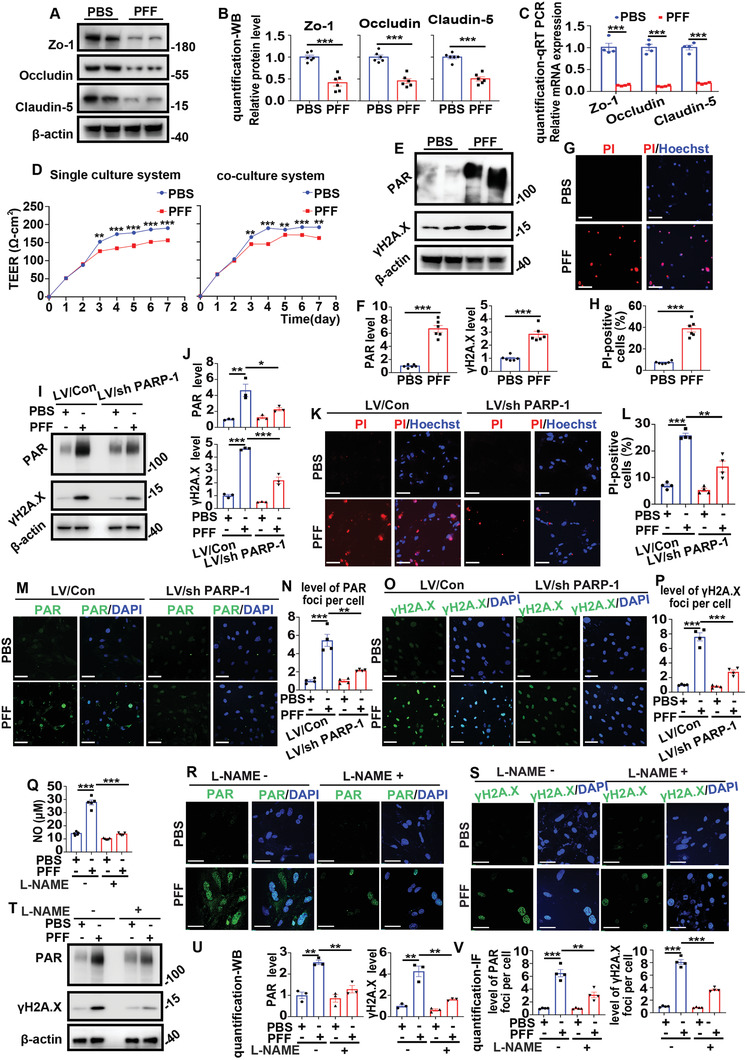
Exogenous *α*‐Syn PFFs impaired epithelial cell barrier permeability and triggered PAR‐driven cell death in BMVECs in vitro. A–H) Experiments for primary BMVECs were treated or pretreated with PBS or 5 µg mL^−1^ *α*‐Syn PFFs for 14 days. A) Representative western blot imaging of ZO‐1, Occludin, and Claudin 5. B) Quantification of (A). ***p < 0.001. The data are expressed as the mean ± SEM. Two‐tailed Student's *t* tests followed by Tukey's post hoc test (six independent replicates). C) qRT‐PCR analysis of the relative mRNA expression of ZO‐1, Occludin, and Claudin 5. ***p < 0.001. The data are expressed as the mean ± SEM. Two‐tailed Student's *t* tests followed by Tukey's post hoc test (four independent replicates). D) Single and coculture systems to detect the TEER value of ECs. The line graph shows the TEER value (Ω cm^2^) of PBS or PFFs on day 1 to day 7. **p < 0.01, ***p < 0.001. The data are expressed as the mean ± SEM. Two‐tailed Student's *t* tests followed by Tukey's post hoc test (three independent replicates). E) Representative western blot images of PAR and *γ*H2A.X. F) Quantification of (E). ***p < 0.001. The data are expressed as the mean ± SEM. Two‐tailed Student's *t* tests followed by Tukey's post hoc test (six independent replicates). G) Representative images of Hoechst and PI staining of BMVECs. H) Quantification of (G). The scatter plots show the percentage of PI‐positive cells. ***p < 0.001. The data are expressed as the mean ± SEM. Two‐tailed Student's *t* tests followed by Tukey's post hoc test (six independent replicates). I–Q) Experiments for primary BMVECs were transfected with control or sh‐PARP‐1 lentivirus after administration of PBS or 5 µg mL^−1^ *α*‐Syn PFFs for 14 days. I) Representative western blot imaging of PAR and *γ*H2A.X. J) Quantification of (I). *p < 0.05, **p < 0.01, ***p < 0.001. The data are expressed as the mean ± SEM. Two‐tailed Student's *t* tests followed by Tukey's post hoc test (three independent replicates). K) Representative images of Hoechst and PI staining. L) Quantification of (K). **p < 0.01, ***p < 0.001. The data are expressed as the mean ± SEM. Two‐tailed Student's *t* tests followed by Tukey's post hoc test (three independent replicates). M) Representative images of PAR and DAPI staining. N) Quantification of (M). The scatter plots show the relative quantities of PAR foci per cell. **p < 0.01, ***p < 0.001. The data are expressed as the mean ± SEM. One‐way ANOVA with Bonferroni's post hoc test (four independent replicates). O) Representative images of *γ*H2A.X and DAPI staining. P) Quantification of (O). The scatter plots show the relative level of *γ*H2A.X foci per cell. **p < 0.01, ***p < 0.001. The data are expressed as the mean ± SEM. One‐way ANOVA with Bonferroni's post hoc test (four independent replicates). Q–V) Experiments for primary BMVECs preincubated with L‐NAME for 12 h and further incubated with *α*‐Syn PFFs for 14 days. Q) Scatter plots showing the NO (nitrite+nitrate) levels. ***p < 0.001. The data are expressed as the mean ± SEM. One‐way ANOVA with Bonferroni's post hoc test (three independent replicates). R) Representative images of PAR and DAPI staining. S) Representative images of *γ*H2A.X and DAPI staining. T) Representative western blot images of PAR and *γ*H2A.X. U) Quantification of (T). **p < 0.01. The data are expressed as the mean ± SEM. One‐way ANOVA with Bonferroni's post hoc test (three independent replicates). V) Quantification of (R) and (S). **p < 0.01, **p < 0.001. The data are expressed as the mean ± SEM. One‐way ANOVA with Bonferroni's post hoc test (three independent replicates).

As shown in **Figure** **7**A–C, the protein and mRNA expression of ZO‐1, Occludin, and Claudin‐5 was significantly decreased after *α*‐Syn PFFs administration. Furthermore, as shown in Figure 7D, *α*‐Syn PFFs administration reduced the TEER value (on day 3 to day 7) in both the coculture system of BMVECs and astrocytes (in vitro BBB model^[^ ^28^ ^]^) and the BMVEC monoculture system.

A study^[^ ^8f^ ^]^ has shown that PARP‐1‐dependent neuronal death induced by *α*‐Syn fibrils leads to neurotoxicity, DNA damage, and *α*‐Syn spreading in neurons. We previously demonstrated in vivo and in vitro that *α*‐Syn fibrils can enter BMVECs and cause impairment of the CMS, NVU coupling, and the BBB. However, whether *α*‐Syn fibrils induce PAR‐driven cell death in BMVECs is unknown. To answer this question, the levels of the PARP‐1 activation product PAR (poly(adenosine 5′‐diphosphate‐ribose)) were measured after the administration of *α*‐Syn PFFs (5 µg mL^−1^ for 14 days) to primary mouse BMVECs. As shown in Figure 7E,F, the expression of PAR increased after *α*‐Syn PFF administration. Subsequently, DNA damage was measured using a monoclonal antibody against a marker of DNA strand breaks, *γ*H2A.X,^[^ ^29^ ^]^ and cell death rates were tested using Hoechst and propidium iodide (PI) staining. The results showed that *α*‐Syn PFF administration induced DNA damage and cell death (Figure 7G,H). Next, PARP‐1 depletion with sh‐PARP‐1 was performed to further determine whether the cell death of BMVECs induced by *α*‐Syn PFFs is PAR driven. As shown in Figure 7I–P, PARP‐1 depletion decreased *γ*H2A.X, PAR levels, and cell death activated by *α*‐Syn PFFs, indicating that the cell death of BMVECs induced by *α*‐Syn PFFs is PAR driven.

We further investigated how *α*‐Syn PFFs cause cell death through PARP‐1. It has been reported that PARP‐1 activation is accompanied by the process of catalyzing the cleavage of nicotinamide adenine dinucleotide (NAD+) into nicotinamide and PAR, which is involved in the repair of DNA damage.^[^ ^30^ ^]^ When pathological stimuli persist in the environment and DNA damage cannot be completely repaired, PARP‐1 is overactivated (e.g., PFF acts as a stimulus to activate PARP‐1 via nitric oxide‐induced DNA damage) and mediates PARP‐1‐dependent programmed cell death in cells.^[^ ^8^, ^31^ ^]^ Therefore, we tested the effect of PFFs on the release of nitric oxide (NO) and the expression of RAR/*γ*H2A.X, and then investigated whether pretreatment with the NO synthase (NOS) inhibitor N*ω*‐nitro‐L‐arginine methyl ester hydrochloride (L‐NAME) could change the effect of PFF on the above indicators (Figure 7Q–V). The results demonstrated that L‐NAME decreased NO release and PAR/*γ*H2A.X expression induced by PFF, indicating that continuous PFF stimulation in BMVECs can cause cell death through NO‐induced DNA damage concomitant with PARP‐1 activation. This is one of the mechanisms that may explain how PFFs in BMVECs cause cell death through PARP‐1.

To collect further evidence regarding the causal relationship between endothelial cell death and neuronal death, two transwell coculture systems, i.e., an ECs‐to‐neurons (E‐N) system and a neurons‐to‐ECs (N‐E) system, were designed^[^ ^9^, ^32^ ^]^ (Figure S7A,B, Supporting Information). As shown in Figure S7C–E in the Supporting Information, the cell death rate of neurons in the “E‐N” system (affected by PFFs) was higher than that of BMVECs in the “N‐E” system in the early, middle, and late stages, indicating that for cell death, the impact of ECs’ *α*‐Syn pathology on neurons (induced by *α*‐Syn PFFs) may be stronger than this impact of neurons’ *α*‐Syn pathology on ECs. Taken together, these results show that *α*‐Syn PFFs cause PAR‐driven cell death in BMVECs in vitro.

### 2.8 Knockout of Lag3 Restored the Damaged Permeability of the Epithelial Cell Barrier and Reduced the PAR‐Driven Cell Death Induced by *α*‐Syn PFFs in BMVECs In Vitro

Next, to further determine whether the damaged epithelial cell barrier permeability and PAR‐driven cell death were caused by *α*‐Syn PFF endocytosis by BMVECs in vitro, Lag3 was knocked out, and we determined whether the abovementioned responses were restored.

**Figure 8:**
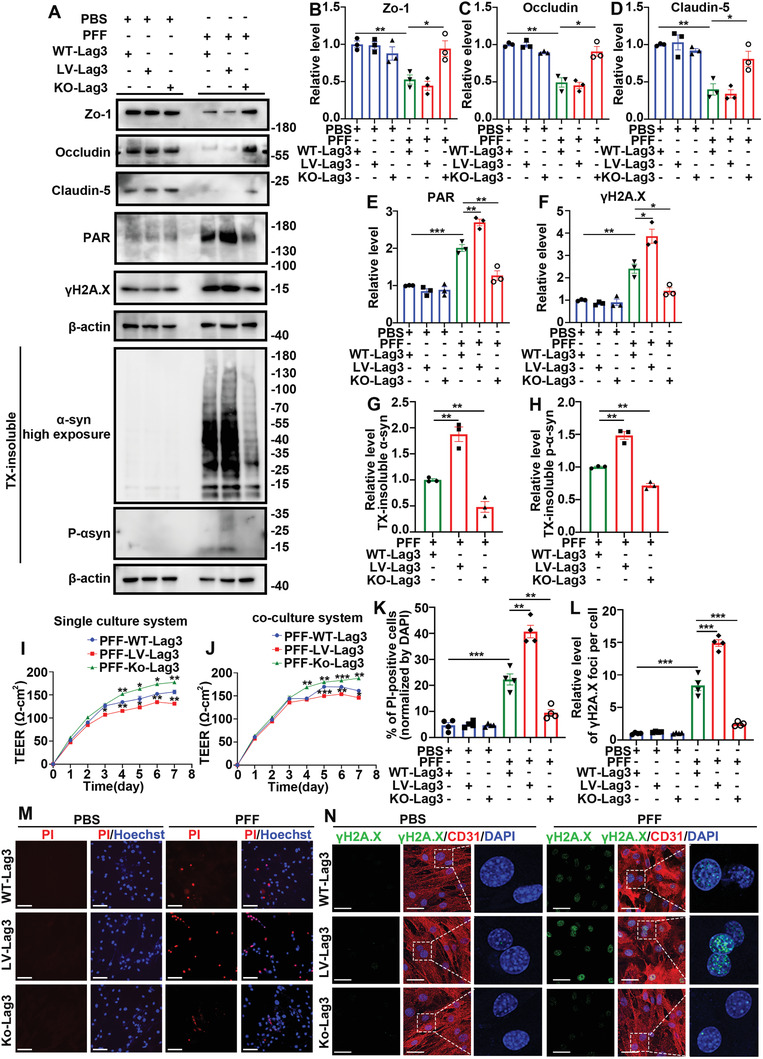
Knockout of Lag3 restored tight junction protein expression and reduced *α*‐Syn PFF‐induced cell death in BMVECs in vitro. Cells were primary BMVECs extracted from WT or Lag3^−/−^ mice or LV‐Lag3‐transfected WT mouse BMVECs (cells from Lag3^−/−^ mice and WT mice were transfected with a negative control lentivirus) treated with PBS or 5 µg mL^−1^ *α*‐Syn PFFs for 14 days. A) Representative western blot images of tight junction proteins (ZO‐1, Occludin, Claudin 5), PAR, *γ*H2A.X, misfolded insoluble *α*‐Syn, and P‐*α*‐Syn. For insoluble fractions, cell lysates were sequentially extracted in 1% TX‐100 (TX‐soluble) followed by 2% SDS (TX‐insoluble) 14 days after PBS or *α*‐Syn PFF treatment. *α*‐Syn PFFs reduced tight junction protein expression, increased PAR‐driven cell death, and initiated insoluble *α*‐Syn inclusions formed in primary cultured BMVECs, which was ameliorated by knockout of LAG3. B–H) Quantification of (A). The scatter plots show the relative levels of tight junction proteins, including B) ZO‐1, C) Occludin, D) Claudin 5, E) PAR, F) *γ*H2A.X, and TX‐insoluble G) *α*‐Syn, H) P‐*α*‐Syn. *p < 0.05, **p < 0.01, ***p < 0.001. The data are expressed as the mean ± SEM. One‐way ANOVA with Bonferroni's post hoc test (three independent replicates). I) Single system and J) coculture system to detect the TEER values of ECs. The line graph shows the TEER value (Ω cm^2^) on day 1 to day 7. *p < 0.05, **p < 0.01, ***p < 0.001. The data are expressed as the mean ± SEM. One‐way ANOVA with Bonferroni's post hoc test (three independent replicates). K) Quantification of (M). The scatter plots show the relative percentage of PI‐positive cells (normalized to DAPI). **p < 0.01, ***p < 0.001. The data are expressed as the mean ± SEM. One‐way ANOVA with Bonferroni's post hoc test (four independent replicates). L) Quantification of (N). The scatter plots show the relative quantities of *γ*H2A.X foci per cell. ***p < 0.001. The data are expressed as the mean ± SEM. One‐way ANOVA with Bonferroni's post hoc test (four independent replicates). M) Representative images of Hoechst and PI staining. N) Representative images of *γ*H2A.X/CD31/DAPI.

Primary BMVECs derived from both Lag3^−/−^ and WT mice and BMVECs overexpressing Lag3 via LV‐Lag3 were used. The cells were exposed to 5 µg mL^−1^ *α*‐Syn PFFs for 14 days. After *α*‐Syn PFF exposure, the protein and mRNA expression of tight junction proteins was examined by western blot analysis and qRT‐PCR. Compared with the primary BMVECs derived from WT mice, BMVECs from Lag3^−/−^ mice had increased expression of ZO‐1, Occludin, and Claudin 5 (**Figure** **8**A–D and Figure S9D–F, Supporting Information). TEER values were subsequently measured to detect the effect of Lag3 on epithelial cell barrier permeability. The TEER values displayed differences on day 3 to day 7. After *α*‐Syn PFFs exposure, overexpression of Lag3 decreased the TEER value, whereas knockout of Lag3 increased the TEER value (Figure 8I,J). These results indicated that knockout of Lag3 in BMVECs restored the reduced tight junction protein expression and damaged epithelial cell barrier permeability induced by *α*‐Syn PFFs.

Further, after *α*‐Syn PFFs exposure, overexpression of Lag3 in cells increased the levels of PAR and *γ*H2A.X, whereas knockout of Lag3 decreased levels of these proteins (Figure 8A,E,F); overexpression of Lag3 also increased the accumulation of pathological insoluble *α*‐Syn and P‐*α*‐Syn, whereas knockout of Lag3 decreased the accumulation of these proteins (Figure 8A,G,H). Immunofluorescence staining with PI/Hoechst and *γ*H2A.X/DAPI was utilized to further verify the regulatory effect of Lag3 on cell death and DNA damage. When primary cultured BMVECs were exposed to *α*‐Syn PFFs (5 µg mL^−1^ *α*‐Syn PFFs for 14 days), overexpression of Lag3 exacerbated cell death, but knockout of Lag3 reduced cell death (Figure 8K,M). Concordantly, with exposure to *α*‐Syn PFFs, the proportions of cells with positive *γ*H2A.X staining were upregulated by Lag3 overexpression and significantly reduced by Lag3 knockout (Figure 8L,N). These results indicated that knockout of Lag3 in BMVECs reduced the PAR‐driven cell death induced by *α*‐Syn PFFs.

### 2.9 Deletion of Endothelial Cell‐Specific Lag3 in C57BL/6 Mice Reversed *α*‐Syn PFFs‐Induced Cognitive Impairment and Cerebral Microvascular Injury

We next investigated whether endothelial cell‐specific Lag3 knockout in vivo prevents *α*‐Syn PFFs from entering ECs and then changes the effects of *α*‐Syn PFFs on cognition and the CMS.

**Figure 9:**
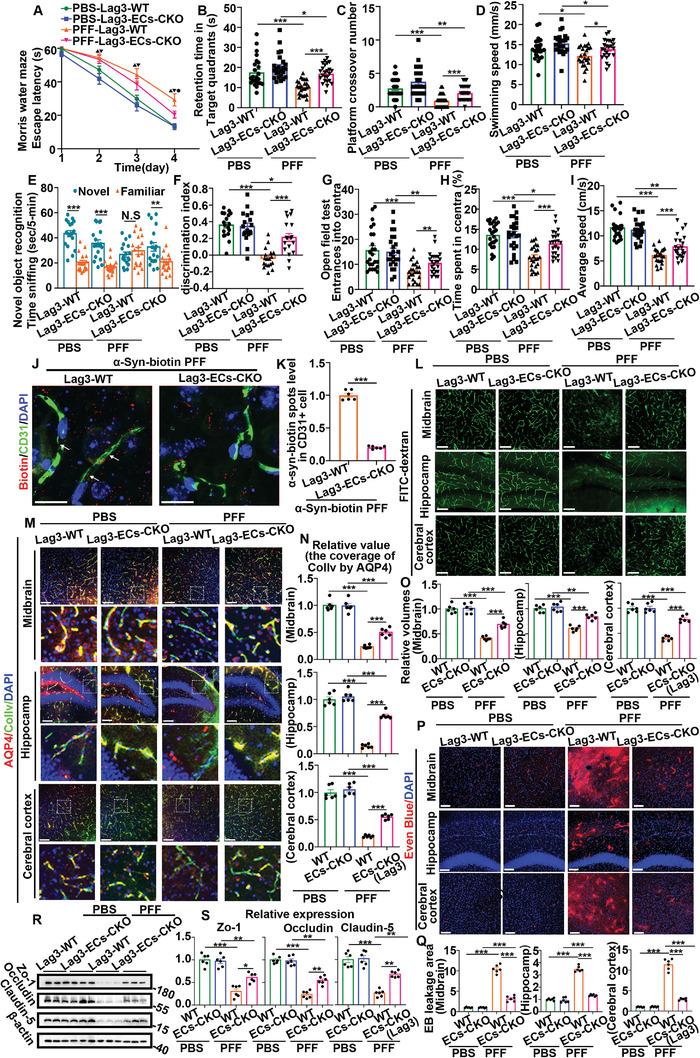
Endothelial cell‐specific Lag3 deletion reduced *α*‐Syn PFF‐induced cognitive impairment and cerebral microvascular injury in vivo. Mice were grouped as follows: PBS‐injected WT mice (PBS‐Lag3‐WT), PBS‐injected Lag3‐ECs‐Cko mice (PBS‐Lag3‐ECs‐Cko), PFF‐injected WT mice (PFF‐Lag3‐WT), and PFF‐injected Lag3‐ECs‐Cko mice (PFF‐Lag3‐ECs‐Cko). 6 months post‐injection was set as the time point to detect cognitive and cerebral microvascular indicators. A–I) Behavioral tests. A–D) MWM test: A) escape latency, ▲ represents PBS‐Lag3‐WT versus PFF‐Lag3‐WT, **p < 0.01 on day 2, ***p < 0.001 on days 3 and 4; ▼ represents PBS‐Lag3‐ECs‐Cko and PFF‐Lag3‐ECs‐Cko, *p < 0.05 on days 2 and 3, **p < 0.01 on day 4; ● represents PFF‐Lag3‐WT versus PFF‐Lag3‐ECs‐Cko, *p < 0.05 on day 4; B) scatter plots showing the time spent in the target quadrant on day 4; C) scatter plots showing the number of platform crossings on day 5; D) scatter plots showing swimming speed on day 5. *p < 0.05, **p < 0.01, ***p < 0.001 (*n* = 24). E,F) NOR test: E) scatter plots showing the time spent sniffing the novel or familiar object out of 5 min during the test period, F) scatter plots showing the discrimination index during the test period. N.S. means no significance, *p < 0.05, **p < 0.01, ***p < 0.001 (*n* = 18). G–I) Open field test: G) scatter plots showing the number of entrances into the central area, H) scatter plots showing the percentage of total time spent in the central area, I) scatter plots showing the average speed (cm s^−1^). *p < 0.05, **p < 0.01, ***p < 0.001 (*n* = 24). All data from behavioral tests are expressed as the mean ± SEM. Differences among multiple means, one‐way ANOVA with Bonferroni's post hoc test; differences between two means, two‐tailed Student's *t* tests followed by Tukey's post hoc test. J) Representative images of biotin/CD31/DAPI of Lag3‐WT versus PFF‐Lag3‐ECs‐Cko mice 14 days after injection with *α*‐Syn PFFs. K) Quantification of (N). The scatter plots show the relative quantities of *α*‐Syn‐biotin punctua in CD31^+^ cells. ***p < 0.001. The data are expressed as the mean ± SEM. Two‐tailed Student's *t* tests followed by Tukey's post hoc test (*n* = 6). L–S) Analysis of cerebral microvascular indicators. L) Representative vessel imaging of FITC‐dextran perfusion. The scale bar denotes 100 µm. O) Quantification of (L). M) Representative immunofluorescence costaining images of AQP4, collagen IV, and merge (AQP4/ColIV/DAPI). The scale bar denotes 100 µm. N) Quantification of (M). P) Representative EB extravasation imaging (EB/DAPI). The scale bar denotes 100 µm. Q) Quantification of (P). R) Representative western blot images of tight junction proteins in midbrain tissues. S) Quantification of (R). All cerebral microvascular indicator data are expressed as the mean ± SEM. **p < 0.5, **p < 0.01, ***p < 0.001. One‐way ANOVA with Bonferroni's post hoc test (*n* = 6).

The MWM, the NOR, and the open field test were applied to assess the cognitive function and behavior of PBS‐injected control mice (PBS‐Lag3‐WT mice), PBS‐injected endothelial cell‐specific Lag3 conditional knockout mice (PBS‐Lag3‐ECs‐Cko mice), PFF‐injected control mice (PFF‐Lag3‐WT mice), and PFF‐injected Lag3‐ECs‐Cko mice (PFF‐Lag3‐ECs‐Cko mice) at 6 months post‐injection. Concordantly, compared with PBS‐Lag3‐WT mice, PFF‐Lag3‐WT mice showed significant cognitive decline and emotional disorder, and although PFF‐Lag3‐ECs‐Cko mice still had inferior outcomes to PBS‐Lag3‐ECs‐Cko mice, they had superior outcomes to PFF‐Lag3‐WT mice (**Figure** **9**A–I and Figure S10A–D, Supporting Information). As shown by these results, deletion of endothelial cell‐specific Lag3 in C57BL/6 mice can reverse *α*‐Syn PFF‐induced cognitive impairment.

Next, *α*‐Syn‐biotin PFFs and unmarked PFFs were injected into Lag3‐ECs‐Cko mice or Lag3‐WT mice to determine whether endothelial cell‐specific Lag3 knockout in vivo reduced the spread of *α*‐Syn PFFs to vascular ECs. Biotin or P‐*α*‐Syn in CD31‐positive vascular ECs was detected around the injection site by immunofluorescence. There was some biotin‐positive signal or P‐*α*‐Syn in CD31‐positive cells in PFF‐Lag3‐WT mice, whereas the levels of biotin or P‐*α*‐Syn in CD31‐positive cells in PFF‐Lag3‐ECs‐Cko mice were barely detectable (Figure 9J,K and Figure S10H,I, Supporting Information). Furthermore, the P‐*α*‐Syn (TX‐insoluble) level was detected by western blotting, and the results were consistent with those of immunofluorescence (Figure S10J–L, Supporting Information). As shown by these results, endothelial cell‐specific Lag3 knockout can reduce the spread of *α*‐Syn PFFs to vascular ECs.

Subsequently, indicators of CMS damage, NVU coupling integrity, and BBB permeability were measured. Regarding CMS damage, compared with PBS‐Lag3‐WT mice, PFF‐Lag3‐WT mice had decreased FITC‐dextran‐labeled perfusion. However, the volumes of FITC‐dextran‐labeled perfusion in PFF‐Lag3‐ECs‐Cko mice were markedly higher than those in PFF‐Lag3‐WT mice (Figure 9L–O). Regarding NVU coupling, compared with PBS‐Lag3‐WT mice, PFF‐Lag3‐WT mice exhibited dramatically reduced colocalization of AQP4 and collagen IV, whereas this colocalization was obviously increased in PFF‐Lag3‐ECs‐Cko mice (Figure 9M,N and Figure S10E–G, Supporting Information). Regarding BBB permeability, the area of EB leakage was much higher in PFF‐Lag3‐WT mice than in PBS‐Lag3‐WT or PFF‐Lag3‐ECs‐Cko mice (Figure 9P,Q). In addition, tight junction proteins were analyzed to evaluate BBB integrity. Consistently, the expression of ZO‐1, Occludin, and Claudin 5 in PFF‐Lag3‐WT mice was much lower than that in PBS‐Lag3‐WT mice and PFF‐Lag3‐ECs‐Cko mice (Figure 9R,S). Based on these results, deletion of endothelial cell‐specific Lag3 in C57BL/6 mice can reverse *α*‐Syn PFFs‐induced cerebral microvascular injury.

In addition, the PAR levels in CD31‐positive cells and the ratio of CD31/BrdU‐positive cells reflecting cell death^[^ ^33^ ^]^ were detected to determine whether endothelial cell‐specific Lag3 knockout in vivo reduced endothelial cell death. As shown in Figure S10J,K,M–P in the Supporting Information, compared with PBS‐Lag3‐WT mice, PFF‐Lag3‐WT mice had higher PAR levels and cell death rates, whereas the PAR levels and cell death rates in PFF‐Lag3‐ECs‐Cko mice were lower than those in PFF‐Lag3‐WT mice. Based on these results, deletion of endothelial cell‐specific Lag3 in C57BL/6 mice can reverse *α*‐Syn PFF‐induced PAR‐driven cell death in ECs.

The above detection of cognitive and microvascular injury indicators revealed no significant differences between the PBS‐Lag3‐WT and PBS‐Lag3‐ECs‐Cko groups, and PFFs still caused cognitive impairment and cerebral microvascular injury in Lag3‐ECs‐Cko mice, indicating that endothelial cell Lag3 is not the only factor controlling cognitive and cerebral microvascular function. Overall, the above results suggest that deletion of Lag3 in ECs in vivo reverses *α*‐Syn PFF‐induced cerebral microvascular injury.

## 3 Discussion

Over recent years, studies have suggested that vascular pathological effects have a definite role in the cognitive impairment of *α*‐synucleinopathies^[^ ^5^ ^]^ and that brain ECs act as bridges that mediate the effects of peripheral factors on cognitive function in aged patients.^[^ ^34^ ^]^ We speculate that there may be an intrinsic link among the pathological effects of *α*‐Syn, pathological effects on the vasculature, and cognitive impairment. In this study, we have revealed the impact of *α*‐Syn fibrils‐related vascular pathological effects on cognitive function in *α*‐synucleinopathies.

As shown in Sections 2.1, 2.2, 2.3, 2.4, compared with AAV‐*α*‐Syn injection, *α*‐Syn PFF injection damages cognitive function and causes mild neuronal damage while leading to more severe cerebral microvascular impairment, NVU coupling disruption, and BBB lesions. Different aggregate species or pathways are induced by AAV and PFF injections. PFF may cause a series of cerebral microvascular impairments by infecting BMVECs, damaging neurons directly or indirectly, and leading to motor and cognitive damage. AAV injection may cause motor and cognitive deficits by activating a strong inflammatory response and accompanying neuron lesions. More specifically, it is noteworthy that different *α*‐Syn aggregate species possess distinct pathogenic mechanisms; AAV injection may tend to produce *α*‐Syn aggregates containing more soluble oligomers, leading to more neuron loss and glial activation, while PFFs injection may tend to act as seeds for nucleation to generate insoluble *α*‐Syn inclusions in BMVECs, leading to more severe cerebral microvascular impairment. We speculate that AAV expression tends to produce more soluble *α*‐Syn aggregates (oligomers), leading to faster, more direct, and more invasive pathological spreading, while PFFs tend to act as seeds for nucleation to generate insoluble *α*‐Syn inclusions, leading to slower, more indirect seeding, and spread of pathology. This insight is also somewhat similar to the previous reports.^[^ ^8^, ^10^, ^35^ ^]^ What is more, this issue needs to be further confirmed in future.

The relationship between neuronal and microvascular injury is a complex problem in *α*‐synucleinopathies. Our results provide evidence supporting their relative independence, but the relationship between microvascular injury and nerve injury cannot be ruled out. For example, crosstalk between neurons and ECs has been reported to play a role in *α*‐Syn pathology.^[^ ^36^ ^]^ Moreover, as indicated in the data of Figure S7 in the Supporting Information, in addition to the effect of neuronal *α*‐Syn pathology on endothelial cell death, the effect of endothelial *α*‐Syn pathology on neuronal cell death is equally important, indicating that the *α*‐Syn pathology of ECs is also important for cognitive impairment of *α*‐synucleinopathies.

Since the cortex and hippocampus are directly involved in cognition, large‐scale neuron loss can directly cause cognitive impairment.^[^ ^37^ ^]^ As shown in the data of Figure S2M,N in the Supporting Information, AAV injection in the cortex and hippocampus tends to produce *α*‐Syn aggregates containing more oligomers than PFFs, which may lead to more significant cortical and hippocampal neuron loss than PFFs,^[^ ^16^ ^]^ resulting in more significant cognitive impairment than PFFs. Nevertheless, it was shown that the AAV and PFF models can both cause significant cognitive damage, and there is great inconsistency between neuronal injury and cerebrovascular injury in the two models. In terms of pathological characteristics, AAV model exhibited more serious neuronal damage than PFF model, while PFF model exhibited more serious cerebral microvascular damage than AAV model. Combining with the cognitive impairment in PFF model, the effect of cerebrovascular injury caused by PFF on cognition is noteworthy. In the next step, the impact of *α*‐Syn PFF‐induced cerebrovascular injury on cognition was investigated. The subsequent experiments with Lag3‐ECs‐Cko mice to specifically block the entry of PFFs into BMVECs provide key evidence for the impact of cerebrovascular injury on cognition.

Next, we further investigate the exact mechanism underlying why *α*‐Syn fibrils cause cerebral cerebrovascular injury. As shown in Section 2.5, *α*‐Syn fibrils can be taken up by BMVECs and transmitted between these cells, providing the insight that BMVECs act as bridges mediating peripheral effects on cognitive function in *α*‐synucleinopathies. In addition, it has been reported that *α*‐Syn fibrils with “prion” properties can act as a “seed,” initiating *α*‐Syn aggregation and inducing cellular pathology, which depends on the cellular level of endogenous *α*‐Syn.^[^ ^38^ ^]^ Furthermore, under physiological conditions, endogenous *α*‐Syn has been reported to be expressed in vascular ECs derived from human brain tissue,^[^ ^39^ ^]^ and we also found baseline endogenous expression of *α*‐Syn in primary mouse BMVECs in vitro. The above evidence supports the idea that CMS is susceptible to *α*‐Syn fibril spreading. As shown in Section 2.6, our research provides evidence for the spreading of *α*‐Syn fibrils to BMVECs and supports the hypothesis that *α*‐Syn fibrils can act as seeds to initiate *α*‐Syn nucleation in BMVECs. These results imply that the mechanism through which *α*‐Syn fibrils damage the cerebral microvasculature may be associated with the series of responses caused by spread of *α*‐Syn fibrils to BMVEC via Lag3‐dependent endocytosis.

However, while small numbers of *α*‐Syn fibrils may enter BMVECs via Lag3‐dependent endocytosis, they do not trigger extensive *α*‐Syn transmission or ultimately damage the CMS. Spreading of *α*‐Syn aggregates in and damage to the CMS may be driven by more complex and specific mechanisms, which deserve further investigation. It has been reported that cell‐to‐cell transmission of *α*‐Syn aggregates is associated with apoptosis and loss of nuclear membrane integrity; the *α*‐Syn aggregates released during cell decomposition can be endocytosed by surrounding cells, causing *α*‐Syn to spread to neighboring cells, implying that the speed at which *α*‐Syn aggregates are released upon cell death and the fibrotic degree of these released *α*‐Syn aggregates determine the extent of *α*‐Syn transmission.^[^ ^40^ ^]^ These findings prompted us to focus on the effects of *α*‐Syn fibrils on cerebral microvascular cell death. As shown in Sections 2.7 and 2.8, our results provide evidence supporting that Lag3‐dependent *α*‐synuclein fibrils endocytosis can induce PAR‐driven cell death in BMVECs. PAR‐driven cell death has been indicated to aggravate the degree of *α*‐Syn fibrosis.^[^ ^8f^ ^]^ Therefore, we speculate that the PAR‐driven cell death caused by *α*‐Syn fibrils is one of the factors that promotes *α*‐Syn reproduction in BMVECs and destroys CMS.

Existing evidence indicates that Lag3 is a key dependent protein for *α*‐Syn PFFs endocytosis, transmission, and toxicity in neurons.^[^ ^27^ ^]^ We have found that Lag3 is also a key protein in endothelial cell uptake of *α*‐Syn PFFs and is involved in inducing cerebral microvascular injury. Although Lag3 is not the only receptor mediating *α*‐Syn PFF endocytosis, after the introduction of *α*‐Syn PFFs for a period of time, Lag3 knockout still reduced the accumulation of intracellular PFFs, which may have been related to the existence of an efflux pathway for *α*‐Syn PFFs.^[^ ^41^ ^]^ Overall, knockout of Lag3 disrupted the balance between endocytosis and efflux of *α*‐Syn PFFs, and ultimately alleviated the pathology of ECs induced by *α*‐Syn PFFs. Knockout of Lag3 in vitro in BMVECs prevents *α*‐Syn PFFs from entering BMVECs, thereby reducing PAR‐driven cell death. As shown in Section 2.9, deletion of Lag3 in ECs in vivo substantially reduces *α*‐Syn PFF‐induced cerebral microvascular injury and cognitive impairment. In short, targeted blockade of *α*‐Syn fibril uptake by ECs alleviates cognitive impairment in mouse models.

## 4 Conclusion

**Figure 10:**
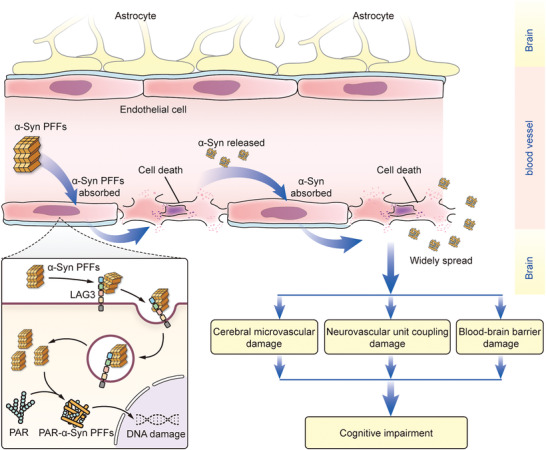
Schematic diagram of the scientific content recorded in our study. Schematic drawing of the relationship among *α*‐Syn fibrils, vascular impairment, and cognitive impairment in *α*‐synucleinopathies.

In conclusion, our study has, for the first time, demonstrated in detail that the effect of *α*‐Syn PFF‐induced cerebral cerebrovascular injury via Lag3‐dependent endocytosis by BMVECs leads to cognitive impairment, which is independent from neuronal damage. This effect may be an important common pathological mechanism that aggravates cognitive impairment in *α*‐synucleinopathies. Specifically, this cerebral microvascular injury is associated with cell responses, including PAR‐driven cell death caused by Lag3‐dependent *α*‐Syn fibril endocytosis by BMVECs. **Figure** **10** shows a schematic diagram summarizing our research findings. Our study provides evidence for targeted blockade of *α*‐Syn fibril uptake by ECs as a strategy to prevent cognitive impairment in *α*‐synucleinopathies.

## 5 Experimental Section

### Preparation of *α*‐Syn PFFs and AAV/2‐A53T

Endotoxin‐free mouse *α*‐syn monomers expressed in *E. coli* were generated with a protocol including high‐speed centrifugation and filtration (for removal of insoluble impurities), precipitation using ammonium sulfate (for removal of impurities), ultrafiltration concentration (for removal of endotoxin particles with larger molecular weights), use of a liquid‐phase endotoxin removal reagent twice, dialysis, anion deposition, and redialysis.

Western blot analysis and TEM were used to detect the structure of the generated *α*‐Syn PFFs (the injection‐fibril length was controlled between 40 and 50 nm) (Figure S8A,B, Supporting Information). Sonication was used to ensure that the majority of fibrils were 50 nm or smaller, which was an important quality control measure.^[^ ^42^ ^]^

Human A53T *α*‐Syn transgene expression was induced using recombinant AAVs (AAV‐A53T; designed by GeneChem, Shanghai, China). The sequence was synthesized according to the methods of Polymeropoulos et al.^[^ ^43^ ^]^

### Construction of Lag3‐ECs‐Cko Mice

As shown in Figure S9A in the Supporting Information, Lag3‐Loxp mice (B6/JGpt‐Lag3^em1Cflox^/Gpt, T009978) were created by a CRISPR/Cas9‐CKO strategy, and Loxp was placed before exon 4 and after exon 8 of Lag3‐201 (Transcript: ENSMUST00000032217.1). To generate Lag3‐ECs‐Cko mice, Lag3‐Loxp mice were then crossed with Tek‐Cre mice (B6/JGpt‐H11^em1Cin(Tek‐icre)^/Gpt, T003764), mice with a mature Cre tool targeting ECs.^[^ ^44^ ^]^ PCR was performed to verify the DNA insertion (Loxp and Tek‐Cre), and western blotting was used to verify whether Lag3 in the endothelial tissue of Cko mice was specifically knocked out. The data are shown in Figure S9B,C in the Supporting Information.

### Animal Experimental Design and Surgical Procedure

Equal numbers of male and female WT C57BL/6 mice (6–8 weeks old) were purchased from the Experimental Animal Center of Southern Medical University. To explore whether *α*‐Syn fibril‐related microvascular pathology affects cognitive function, the mice were divided into four groups: healthy controls (the control group), mice that received unilateral injections of negative control AAV/2 (the sham group), mice that received unilateral injections of AAV/2‐A53T (the AAV‐A53T group), and mice that received unilateral injections of mouse *α*‐Syn PFFs (the PFF group). 6 months post‐injection was set as the time point to detect cognitive and cerebral microvascular indicators. To investigate the effect of blocking *α*‐Syn PFF uptake by ECs on cerebral microvascular injury and cognitive impairment, Lag3‐ECs‐Cko mice were used.

According to the Mouse Brain in Stereotaxic Coordinates atlas (George P and Keith B), injections were made into the left SN, hippocampus, and cerebral cortex at the following coordinates (in mm): SN: anteroposterior (AP), −3.08; mediolateral (ML), dorsoventral (DV), −4.5; hippocampus: AP, −2.5; ML, 1.68; DV, −1.8; and cerebral cortex: AP, 0.0; ML 1.5, DV, −1.6; mm. A total of 1.8 ×10^10^ genome copies (6.02 × 10^12^ genome copies mL^−1^, 1.5 µL per site) and 20 µg of human or mouse *α*‐Syn PFFs (5 µg µL^−1^, 2 µL per site) were injected per animal.

The animal experiment conformed to the guidelines of the National Institutes of Health Guide for the Care and Use of Laboratory Animals (NIH Publications No. 8023, revised 1978) and complied with the ARRIVE guidelines. All animal experiments were approved by the Research Ethics Committee of Guangdong Provincial People's Hospital (GDREC2019133A).

### Behavioral Experiments

1) *Rotarod test*: As previously described,^[^ ^45^ ^]^ after 3 days of training, the mice underwent a four‐trial test (with at least 20 min between each trial) under an accelerating protocol starting at 4 rpm and reaching 40 rpm in 5 min, and the latency to fall (s) was recorded to reflect the motor coordination and balance of animals. 2) *Pole test*: As previously described,^[^ ^46^ ^]^ mice were placed head‐upward on the top of a vertical rough‐surfaced pole (diameter 8 mm; height 55 cm), and the time until the mouse descended to the floor (locomotor activity time) was recorded, with a maximum duration of 120 s. 3) *NOR test*: As previously described,^[^ ^47^ ^]^ briefly, only the mice without innate side preference were included in the formal NOR test after a 1 week adaptation period. In the training and testing period, each mouse was habituated to a novel arena and then given a 10 min familiarization session with two identical objects. After familiarization, the mouse was removed from the testing arena. After a 1 h intertrial interval, the mouse was placed back into the arena with one familiar object and one novel object. The time spent investigating both objects was recorded. The time spent sniffing (s/5 min) the familiar or novel object and the discrimination index were determined as indicators of recognition memory. 4) *MWM and open field test*: According to previous study,^[^ ^12^ ^]^ the MWM and open field test were performed with a real‐time video tracking system, and the results were analyzed with EthoVision XT 7.0 software (Wageningen, Netherlands).

### qRT‐PCR

Total RNA was extracted using RNAiso Plus Reagent (TaKaRa, 9108, Japan) and reverse‐transcribed into cDNA, and mRNA expression was determined utilizing a SYBR Green PCR kit (Bio‐Rad, 6572, USA). The data were analyzed using the 2^−ΔΔCT^ method. The primer sequences are listed in Table S2 in the Supporting Information.

### Pathological Analysis of Brain Tissue

The experimental animals were sacrificed after behavioral tests for subsequent pathological analysis. 1) *Measurement of cerebral microvascular density*: To measure the cerebral microvascular density in the brain, 2.5% FITC‐conjugated dextran (0.1 mL kg^−1^, Sigma‐Aldrich, Saint Louis, MO, USA) was administered via tail vein injection. Measurement of FITC‐dextran in vessels can be used to identify capillaries. Decapitation was performed more than 10 min after FITC‐dextran administration to allow FITC‐dextran to fully penetrate into the brain parenchyma. Frozen coronal sections (60 µm; Leica SM, Germany) of the midbrain, hippocampus, and cortex were prepared to observe the vessel density. The results were analyzed with a laser‐scanning confocal microscope (Leica, Germany). The vascular volume ratio was calculated with software (BitPlane AG, Switzerland). The volume and diameter of classified vessels (vein and artery) were assessed with software (https://imagej.nih.gov/ij/plugins/vesselj/index.html). 2) *Preparation for TEM and assessment of results*: After anesthesia, the mice were perfused with normal saline through the left ventricle. Starting from the injection site the brain tissue was harvested distally at a distance of 2 mm. The volume of a single brain tissue sample was ≈1.5 mm^2^, and the tissues were marked as segment‐1 (S‐1), segment‐2 (S‐2), and segment‐3 (S‐3). The tissues were removed and soaked in special fluid for electron microscopy (Spectrum Chemical, Gardena, CA, USA) overnight at 4 °C. After being washed with PBS three times for 15 min (pH 7.4), the brain tissues were further fixed in 0.5% osmium tetroxide solution for 1 h. Next, propylene oxide was infused into the brain tissues three times for 15 min each, and the tissues were embedded in epoxy resin (Structure Probe Inc., USA). A detailed analysis of ECs was performed by TEM. Quantitative indicators of the basement membrane and tight junctions were measured to reflect the damage to the capillary ultrastructure. The statistical indicator of the damage to the basement membrane was the proportion of IBM, TBM, and DBM; the statistical indicator describing the damage to tight junctions was the ratio of ITJs to DTJs.^[^ ^18^ ^]^ 3) *Measurement of in vivo BBB permeability*: As previously described,^[^ ^24^ ^]^ EB and two sizes of FITC‐fluorescence‐labeled dextran dye were used to determine the in vivo permeability of the BBB. Briefly, 3% dye (45 mg kg^−1^, Sigma, St. Louis, MO, USA) was injected intravenously into the mice and allowed to circulate for 3 h, after which the mice were anesthetized and transcardially perfused with 0.9% NaCl. For leakage area obversion, the mouse brains were collected and processed to generate frozen slices (the slice thickness was 15 µm). EB (red EB fluorescence was measured at 632 nm) and FITC‐fluorescence extravasation in the midbrain, hippocampus, and cerebral cortex was observed using a confocal microscope (Leica, Germany). For quantitation of EB leakage, brain tissues were immersed in formamide (500 µL, Sigma, St. Louis, MO, USA) at 60 °C for 24 h. The tissues were then centrifuged (12 000 × *g*, 10 min) at 4 °C. The centrifuged supernatants were measured at 620 and 740 nm in a spectrophotometer capable of reading at two wavelengths simultaneously. The total EB leakage (µg g^−1^ tissue) was calculated from its standard curve as a measure of the BBB permeability change. 4) *Western blot analysis*: Brain tissues and cells were lysed in radioimmunoprecipitation assay buffer. The protein content was determined using a Pierce BCA protein assay. Next, the supernatants were evenly mixed with gel loading buffer at a ratio of 1:1, boiled for 10 min, subjected to SDS‐PAGE, and then transferred onto polyvinylidene fluoride membranes (Millipore, USA). Next, the membranes were incubated with primary antibodies overnight at 4 °C and then incubated with horseradish peroxidase (HRP)‐conjugated secondary antibodies. HRP activity was detected using enhanced chemiluminescence detection reagent, and the bands were captured with the Bio‐Rad ChemiDoc MP system and quantified with ImageJ software. 5) *Tissue processing and immunofluorescence*: Brains were dissected and postfixed for 24 h in 4% paraformaldehyde or 10% trichloroacetic acid at room temperature for immunostaining. The samples were then embedded in Tissue‐Tek O.C.T. compound, frozen on dry ice, and incubated with primary antibodies overnight at 4 °C. After primary antibody incubation, the samples were washed with PBS‐T three times and incubated with a secondary antibody for 1 h at room temperature. 6) *Immunocytochemistry and quantification of TH^+^ and NeuN^+^ cell numbers*: An unbiased stereological counting method was used.^[^ ^48^ ^]^ Beginning at the same starting point for every mouse in each group, a total of six sections (30 µm thick) through the substantia nigra (every 6th section evenly spaced at 180 µm) within the range covering the whole substantia nigra pars compacta (SNc) (between −2.8 and −3.88 mm AP from the bregma) were processed for TH and NeuN immunohistochemistry. Quantitative analyses were performed using computer‐assisted stereology with a Leica DM6 photomicroscope equipped with a digital camera and StereoInvestigator software (MicroBrightfield Williston VT). Unilateral counts of TH^+^ and NeuN^+^ cells in the SNc were performed at regular predetermined intervals (the counting frame size was 80 µm × 80 µm, and the sampling grid size was 100 µm × 100 µm). The coefficient of error was calculated as an estimate of precision, and values under 0.1 were accepted.

The antibodies used in this study are shown in Table S3 in the Supporting Information.

### HTRF *α*‐Syn Aggregation Detection

The HTRF‐assay was established based on the concept of FRET, and *α*‐Syn oligomers were theoretically believed to be detected more sensitively than other aggregate species in FRET‐based assays.^[^ ^17^, ^49^ ^]^ The soluble *α*‐Syn aggregations in different models were measured with an HTRF *α*‐Syn aggregation kit (PerkinElmer #6FASYPEH). First, the anti‐*α*‐Syn‐Tb‐Cryptate and anti‐*α*‐Syn‐d2 antibodies in this kit were separately applied to immunoblotting to detect human *α*‐Syn and mouse *α*‐Syn recombinant proteins; the results showed that both antibodies recognize *α*‐Syn proteins of different species without specificity. Then, tissue homogenates of the midbrain, hippocampus, and cerebral cortex from different models were, respectively, applied to the HTRF *α*‐Syn aggregation kit, all following the manufacturer's instructions (the quality of detection in each group was adjusted by western blotting to make the level of total *α*‐Syn protein in the same brain region consistent). Finally, the FRET signals were measured by a recommended plate reader (Thermo Scientific, Varioskan LUX) with the excitation wavelength at 380 nm and emission wavelength at 665/620 nm.

### Preparation of *α*‐Syn‐Biotin PFF and Alexa Fluor 488‐Tagged *α*‐Syn PFFs

The *α*‐Syn‐biotin PFFs were synthesized using an EZ‐Link Sulfo‐NHS‐LC‐Biotin kit (Thermo Fisher) according to the methods of Mao et al.,^[^ ^27^ ^]^ and an Alexa Fluor kit (Invitrogen) was used to label exogenous mouse PFFs (Alexa Fluor 488‐tagged *α*‐Syn PFFs), as previously shown.^[^ ^2a^ ^]^

### Endosome Enrichment for Tissues

Neural tissue or cerebral microvascular tissue was isolated,^[^ ^50^ ^]^ and the protein was extracted from the isolated tissue (proteins from three mice were mixed into one protein sample). Then, an Endosome Isolation and Cell Fractionation Kit (Invent Biotechnologies) was used to obtain the endosome‐enriched protein.^[^ ^51^ ^]^ Platelet endothelial cell adhesion molecule (PECAM, specifically expressed in BMVECs) or TUJ‐1 (specifically expressed in neurons) antibodies were used in combination to detect the purity of isolated neuronal tissue or brain microvascular tissue.

### Cell Surface Binding Assay

As previously described,^[^ ^27^ ^]^ the dissociation curve of *α*‐Syn‐biotin PFF binding to primary BMVECs was assessed. Quantification was performed with ImageJ, and the area values with different concentrations of a‐Syn‐biotin PFFs (*α*‐syn‐biotin monomer equivalent (× 10^−9^ m), concentrated in 0.1% TX‐100 conditions) were input into the Prism program to obtain the disassociation constant.

### Cultivation of Primary Mouse BMVECs

Primary mouse BMVEC cultivation was performed as previously described.^[^ ^52^ ^]^ Briefly, the brain tissues of 1 to 2 weeks old mice were physically fragmented and then separated by gradient centrifugation and chemical enzyme digestion to obtain cells for culture, which were then cultured in endothelial cell medium (endothelial growth medium containing 5% serum and growth supplements). Flow cytometry and immunofluorescence for CD31 immunoreactivity were used to validate the BMVEC culture (Figure S5B,C, Supporting Information).

### Measurement of TEER Values

Two systems, including a noncontact coculture system of primary BMVECs and primary astrocytes (an in vitro BBB model^[^ ^28^ ^]^) and a separate primary BMVEC culture system, were used to detect epithelial cell barrier integrity and permeability. For the coculture system, a transwell chamber with a pore size of 0.4 µm was used to separate BMVECs and astrocytes. BMVECs pretreated with 5 µg mL^−1^ *α*‐syn PFFs for 14 days were seeded on the microporous membrane of the transwell chamber coated with 1% gelatine. The primary astrocytes were seeded in the outer pool coated with poly‐L‐lysine beforehand. The TEER value was measured with a TEER instrument (Millipore MERS00002) for 7 days.

### Cell Transfection and Lentivirus Production

Cells were infected with lentivirus carrying the Lag3 overexpression construct and lentiviral particles carrying three shRNAs targeting Lag3 (Figure S8C,D, Supporting Information). These lentiviruses were generated by Hanbio Biotechnology Co., Ltd. (Shanghai, China). Detailed sequence information can be found in the Methods section in Table S4 in the Supporting Information. sh‐PARP‐1 and control lentivirus vectors were purchased from Santa Cruz.

### Co‐IP

Cells were collected after 7 days of *α*‐Syn PFFs treatment and lysed with cell lysis buffer. The lysates were mixed with streptavidin‐AP or anti‐Lag3 antibody or incubated overnight at 4 °C. Then, preblocked agarose beads were added, and the mixture was incubated for 2 h at 4 °C. The product was washed with 1 mL of lysis buffer three times. Then, the beads were eluted for western blotting. The targeted proteins were examined with anti‐Lag3 or anti‐*α*‐syn antibodies.

### NO Measurement and Hoechst and PI Staining

As previously described,^[^ ^8f^ ^]^ NO levels were measured using an NO assay kit (Abcam, ab65328, MA) according to the manufacturer's instructions. Cell death was measured by staining with 10 × 10^−6^ m Hoechst 33342 (Sigma, 14533, USA) and 5 × 10^−6^ m PI (DojinDo, C542, Japan) at *λ*_ex_ = 530 nm and *λ*_em_ = 580 nm. Images were taken using a confocal microscope (Leica, Germany), and the percentage of PI‐positive cells was quantified with ImageJ software

### Validation of Lag3 Antibody Specificity

To determine the specificity of the Lag3 antibody, Lag3 staining was also performed in primary neurons (as positive controls) and SH‐SY5Y cells (as negative controls)^[^ ^27^ ^]^ (Figure S8E, Supporting Information). The results showed that there was a Lag3 signal in primary neurons and BMVECs but not in SH‐SY5Y cells. Then, Lag3 staining was performed in BMVECs treated with lentivirus (for overexpression of Lag3) or three shRNAs (for knockdown of Lag3) (Figure S8F,G, Supporting Information).

### Statistical Analysis

All data were analyzed with GraphPad Prism 7 software and were expressed as the mean ± SEM. One‐way ANOVA followed by post hoc Bonferroni's test was conducted to analyze multiple independent results. Differences between two means were assessed by unpaired two‐tailed Student's *t* test followed by Tukey's post hoc test. Power analysis was conducted using Power and Sample Size Calculations Software to ensure reasonable statistical power.^[^ ^53^ ^]^ A value of *p* < 0.05 was considered to indicate statistical significance.

## Conflict of Interest

The authors declare no conflict of interest.

## Supporting information

Supporting Information

## Data Availability

The data that support the findings of this study are available from the corresponding author upon reasonable request.
