# Supplementary material for: Cerebral Microvascular Injury Induced by Lag3‐Dependent α‐Synuclein Fibril Endocytosis Exacerbates Cognitive Impairment in a Mouse Model of α‐Synucleinopathies
Source: Adv Sci (Weinh). 2023 Jun 28;10(25):2301903. doi: 10.1002/advs.202301903 (PMC10477873; doi:10.1002/advs.202301903)
Supplement: Supplementary file 1 — Supporting Information [file ADVS-10-2301903-s001.pdf]

ADVANCED SCIENCE
Open Access

Supporting Information

for *Adv. Sci.*, DOI 10.1002/advs.202301903

Cerebral Microvascular Injury Induced by Lag3-Dependent α-Synuclein Fibril Endocytosis Exacerbates Cognitive Impairment in a Mouse Model of α-Synucleinopathies

Qingxi Zhang, Qingrui Duan, Yuyuan Gao, Peikun He, Rui Huang, Haifeng Huang, Yanyi Li, Guixian Ma, Yuhu Zhang, Kun Nie and Lijuan Wang**

Supplementary materials

Fig. S1

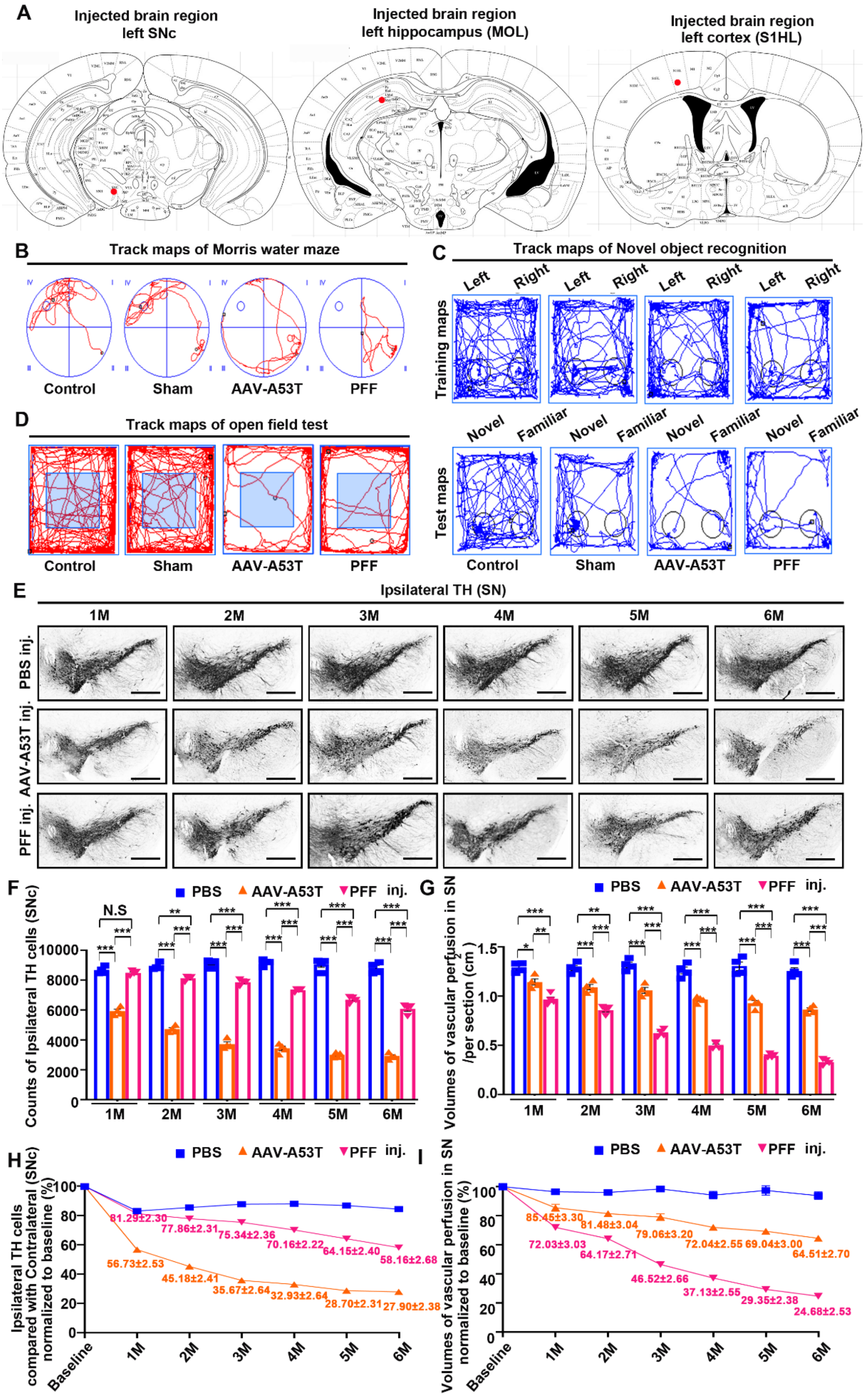

Fig. S1 Determination of the time point of cognitive decline and cerebral microvascular injury in C57BL/6 mice caused by AAV-A53T or α-Syn PFFs.

(A) The three injected brain regions (marked with red dot)， as shown in a mouse brain atlas (http://labs.gaidi.ca/mouse-brain-atlas/, coronal plane), were the substantia nigra pars compacta (SNc), the molecular layer of the hippocampal dentate gyrus (MOL), and the primary somatosensory cortex (S1HL). (B) Representative track maps of the MWM test for each group. (C) Representative track maps of the open field test for each group. (D) Representative track maps in the training and test periods of the NOR test for each group. (E) Representative immunohistochemical staining for TH in the SN of WT mice at 1, 2, 3, 4, 5 and 6 months after PBS, AAV-A53T, and α-Syn PFF injection. The scale bar denotes 500 μm. (F) Scatter plots showing the counts of ipsilateral TH cells in the SNc of WT mice at 1, 2, 3, 4, 5 and 6 months after PBS, AAV-A53T, and α-Syn PFF injection. N.S means no significance, **P<0.01, ***P<0.001. The bars represent the mean ± SEM. One-way ANOVA by Bonferroni's post hoc test. (n=4). (G) Scatter plots showing the volumes of FITC-dextran-marked perfusion in the SN/per section (cm^2) for WT mice at 1, 2, 3, 4, 5 and 6 months after PBS, AAV-A53T, and α-Syn PFF injection. **P<0.01, ***P<0.001. The bars represent the mean ± SEM. One-way ANOVA by Bonferroni's post hoc test. (n=4). (H) Line graph showing the relative percentage of ipsilateral compared with contralateral TH^+ cells (SNc) normalized to the baseline of WT mice at 1, 2, 3, 4, 5 and 6 months after PBS, AAV-A53T, and α-Syn PFF injection. (I) Line graph showing the relative percentages of the volumes of FITC-dextran-marked perfusion normalized to the baseline of WT mice at 1, 2, 3, 4, 5 and 6 months after PBS, AAV-A53T, and α-Syn PFF injection.

Fig. S2

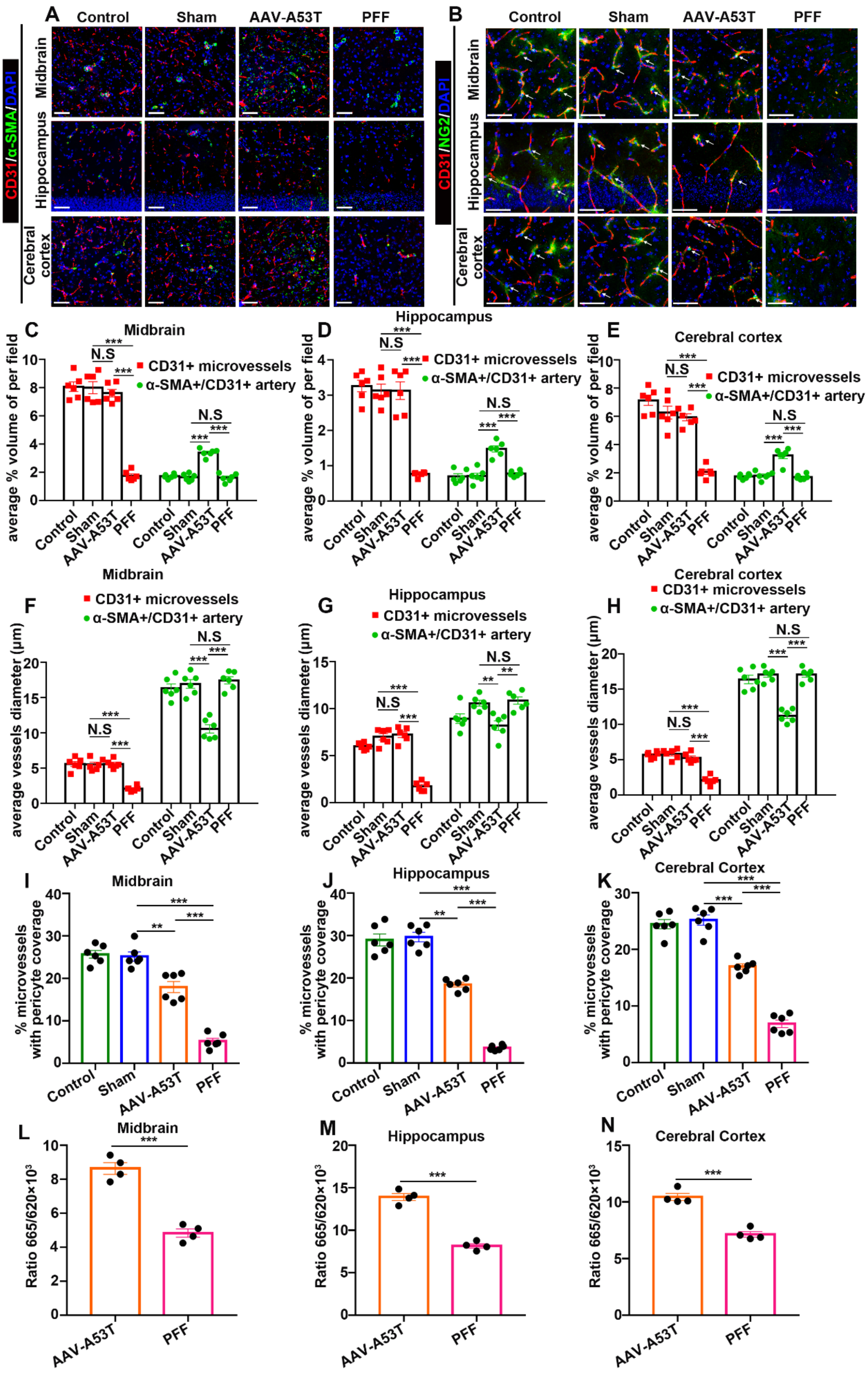
A
Control
Sham
AAV-A53T
PFF
CD31/α-SMA/DAPI
Midbrain
Hippocampus
Cerebral cortex
B
CD31/NG2/DAPI
C
Midbrain
CD31+ microvessels
α-SMA+/CD31+ artery
average % volume of per field
D
Hippocampus
E
Cerebral cortex
F
G
H
average vessels diameter (μm)
I
J
K
Cerebral Cortex
% microvessels with pericyte coverage
L
M
N
Ratio 665/620×10³

Fig. S2 Assessment of the volume and diameter of vessels and microvascular pericyte coverage in each group. (A) Representative immunofluorescence costaining images of CD31 and α-SMA in the midbrain, hippocampus and cerebral cortex for each group. The scale bar denotes 50 μm. (B) Representative immunofluorescence costaining images of CD31 and NG2 in the midbrain, hippocampus and cerebral cortex for each group. The scale bar denotes 50 μm. (C~E) Scatter plots showing the percentage of average volume per field for classified $CD31^+$ microvessels or α-SMA^+/$CD31^+$ arteries in the midbrain (C), hippocampus (D) and cerebral cortex (E) for each group. N.S means no significance, ***P<0.001. The data are expressed as the mean ± SEM. One-way ANOVA by Bonferroni's post hoc test (n=6). (F~H) Scatter plots showing the average vessel diameter for classified $CD31^+$ microvessels or α-SMA^+/$CD31^+$ arteries in the midbrain (F), hippocampus (G) and cerebral cortex (H) for each group. N.S means no significance, **P<0.01, ***P<0.001. The data are expressed as the mean ± SEM. One-way ANOVA by Bonferroni's post hoc test (n=6). (I~K) Scatter plots showing the percentages of microvessels with pericyte coverage in the midbrain (I), hippocampus (J) and cerebral cortex (K) for each group. **P<0.01, ***P<0.001. The data are expressed as the mean ± SEM. One-way ANOVA by Bonferroni's post hoc test (n=6). (L~N) The amounts of α-Syn aggregates in homogenates of the midbrain (L), hippocampus (M) and cerebral cortex (N) from AAV or PFF models, quantified using homogenous time-resolved fluorescence (HTRF) (total α-Syn protein in the same brain region of each group were at uniform levels), expressed as Ratio $665/620\times10^3$. ***P<0.001. The data are expressed as the mean ± SEM. Two-tailed Student's t test followed by Tukey's post hoc test (n=4).

Fig. S3

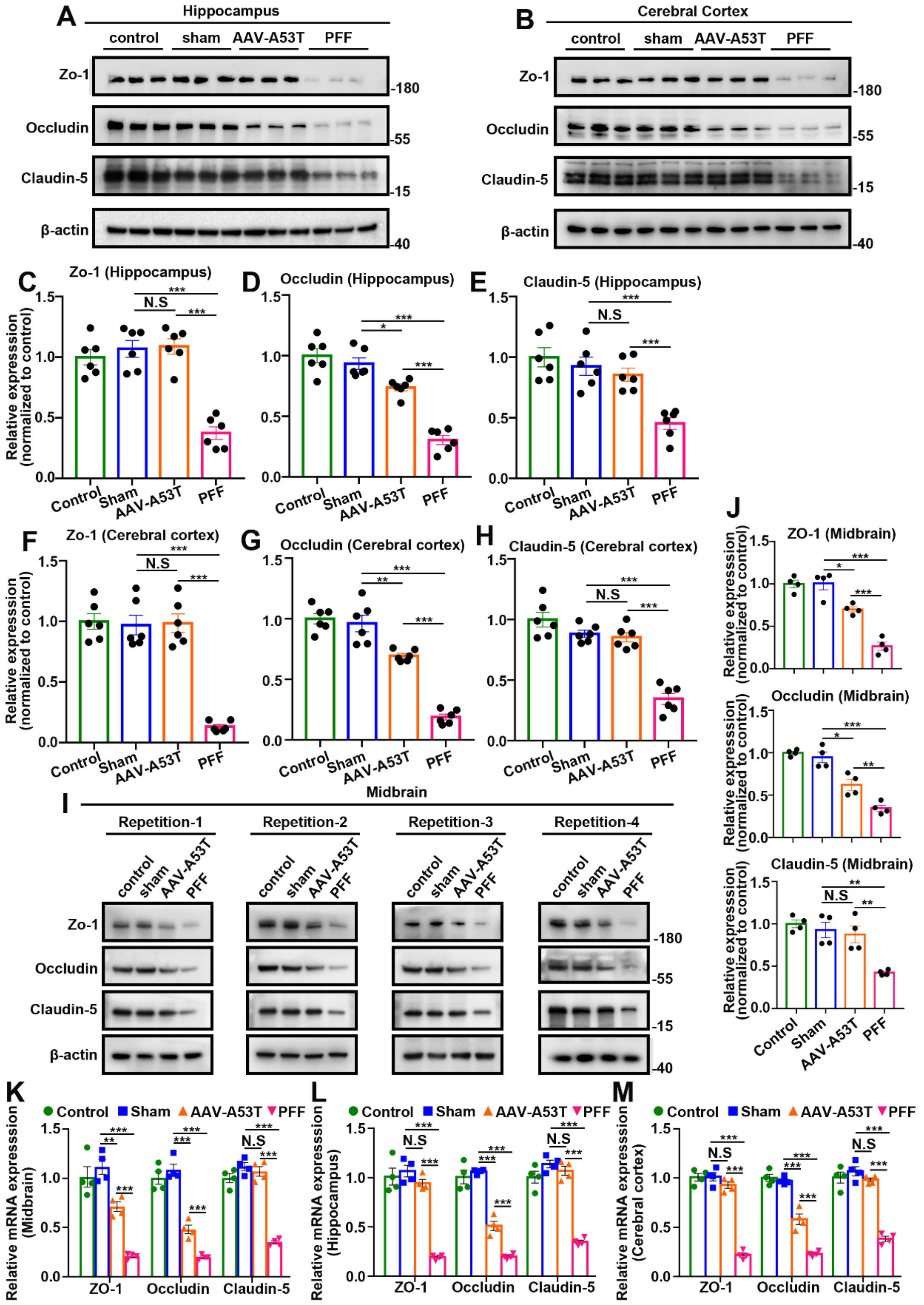

Fig. S3 Measurement of tight junction protein expression in each group. (A) Representative western blot imaging of tight junction proteins in hippocampal tissues. (B) Representative western blot imaging of tight junction proteins in cerebral cortex

tissues. (C~E) Quantification of (A). The scatter plots show the relative expression of tight junction proteins in hippocampal tissues (normalized to control), including Zo-1 (C), Occludin (D), and Claudin-5 (E). N.S means no significance, *P<0.05, ***P<0.001. The data are expressed as the mean ± SEM. One-way ANOVA by Bonferroni's post hoc test (n=6). (F~G) Quantification of (B). The scatter plots show the relative expression of tight junction proteins in cerebral cortex tissues (normalized to control), including Zo-1 (F), Occludin (G), and Claudin-5 (H). N.S means no significance, **P<0.01, ***P<0.001. The data are expressed as the mean ± SEM. One-way ANOVA by Bonferroni's post hoc test (n=6). (I) Four independent results of the measurement of tight junction proteins in midbrain tissues by western blotting. (J) Quantification of (I). N.S means no significance, *P<0.05, **P<0.01, ***P<0.001. The data are expressed as the mean ± SEM. One-way ANOVA by Bonferroni's post hoc test (n=4).

Fig. S4

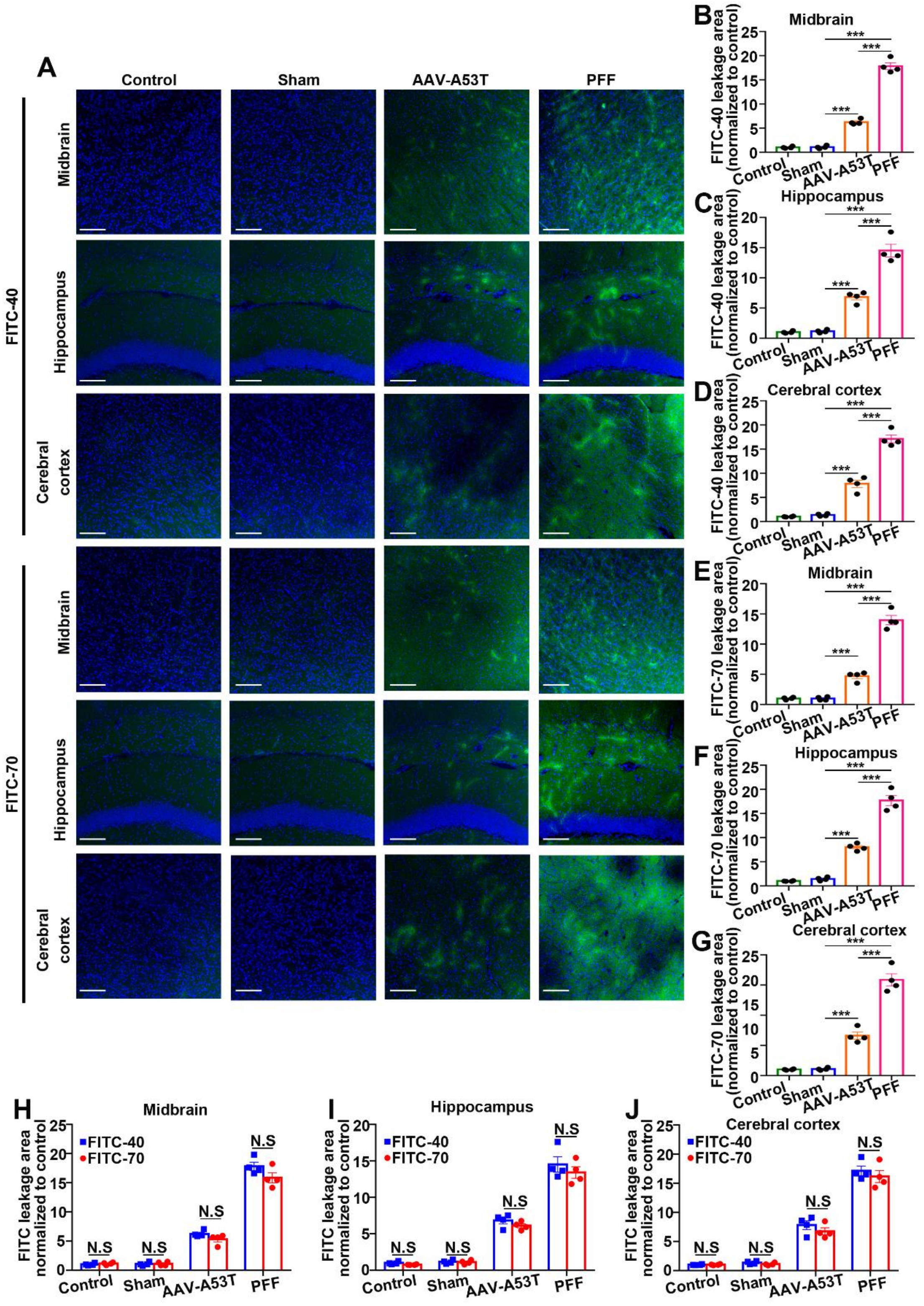

Fig. S4 Measurement of *in vivo* BBB permeability with 40-kDa and 70-kDa fluorescence-labelled dextran in each group. (A) Representative 40-kDa and 70-kDa fluorescence-labelled dextran extravasation imaging in the midbrain, hippocampus, and

cerebral cortex for each group; the scale bar denotes 100 μm. (B~D) Scatter plots showing the relative 40-kDa fluorescence-labelled dextran leakage area (normalized to control) in the midbrain (B), hippocampus (C), and cerebral cortex (D) for each group. ***P<0.001. The data are expressed as the mean ± SEM. One-way ANOVA by Bonferroni's post hoc test (n=6). (B~D) Scatter plots showing the relative 70-kDa fluorescence-labelled dextran leakage area (normalized to control) in the midbrain (E), hippocampus (F), and cerebral cortex (G) for each group. ***P<0.001. The data are expressed as the mean ± SEM. One-way ANOVA by Bonferroni's post hoc test (n=6). (H~J) Scatter plots showing the relative 40-kDa and 70-kDa fluorescence-labelled dextran leakage areas (normalized to control, 40-kDa fluorescence) in the midbrain (H), hippocampus (I), and cerebral cortex (J) for each group. N.S means no significance. The data are expressed as the mean ± SEM. Two-tailed Student's t test followed by Tukey's post hoc test (n=6).

Fig. S5

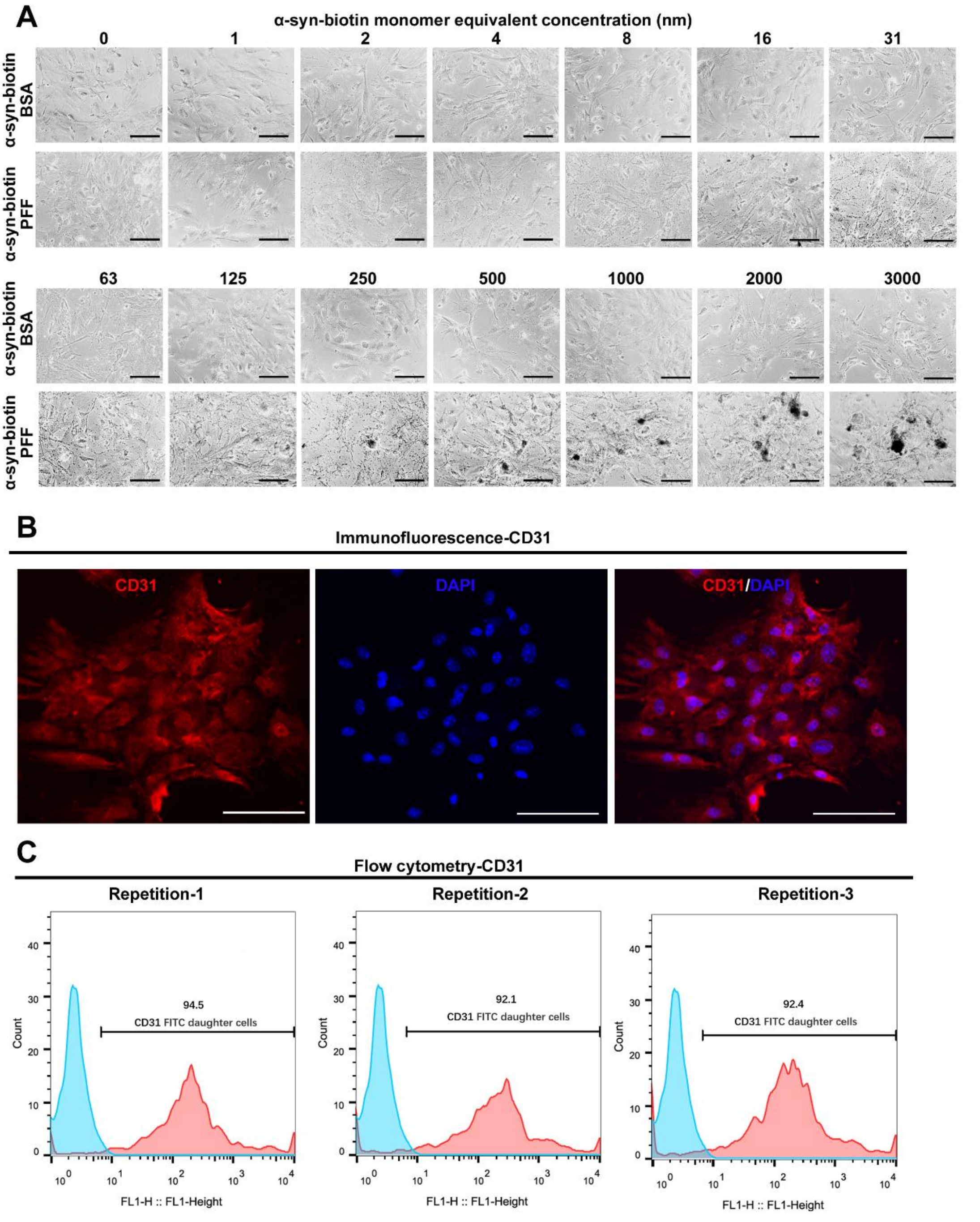

Fig. S5 Binding images and validation of BMVEC culture. (A) Representative streptavidin- alkaline phosphatase (AP) staining marking α-Syn-biotin bound to the cell surface. The concentrations of α-Syn-biotin PFF were established with α-Syn-biotin monomer equivalents (nM) in 0.1% TX-100 conditions. (B~C) Immunofluorescence (B) and flow cytometry (C) for CD31 immunoreactivity to validate the BMVEC culture. The scale bar denotes 200 μm.

Fig. S6

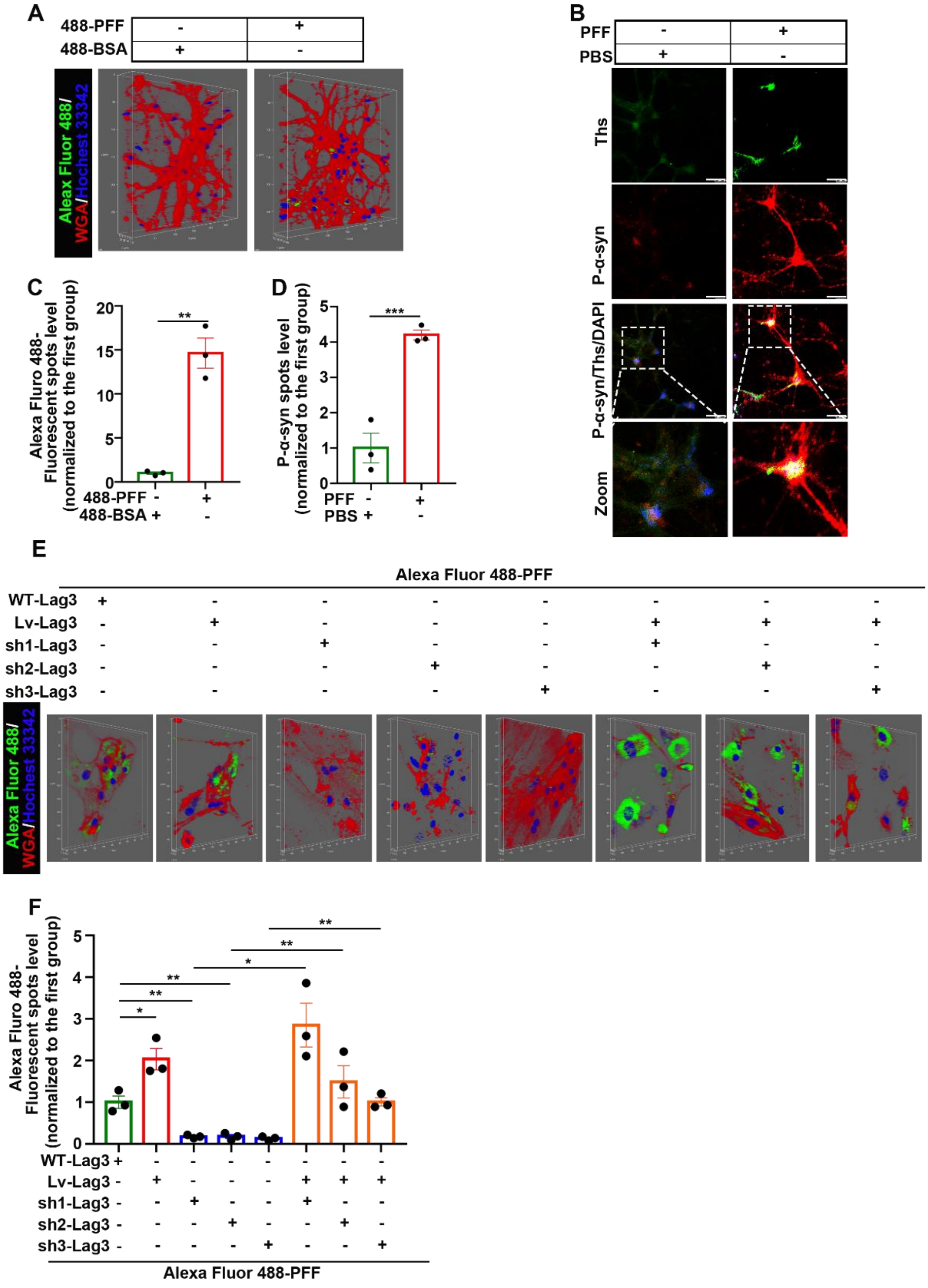

Fig. S6 Detection of exogenous α-Syn PFFs in primary neurons and the effect of LV-Lag3 or sh-Lag3 in BMVECs. (A) Representative confocal 3D imaging of live cell staining to capture images of exogenous α-Syn PFFs entering primary neurons. The cells were treated with 488-PFF or 488-BSA for 48 hours. Alexa Fluor 488-tagged α-Syn PFFs were used to track exogenous α-Syn PFFs (Alexa Fluor 488-tagged BSA was

used as a negative control), Alexa Fluor 555-WGA dye was used as a cytoskeleton marker, and Hoechst 33342 was used to stain nuclei. The cells were washed with PBS 3 times to remove the free biotin. (B) Representative costaining images of P-α-Syn and ThS in primary neurons treated with 488-PFF or 488-BSA for 14 days. The colocalization of P-α-Syn and ThS was clearly observed, as indicated by the white arrows. The scale bar denotes 100 μm. (C) Quantification of (A). The scatter plots show the relative Alexa Fluor 488 fluorescent spot levels (normalized to the first group). **P<0.01. The data are expressed as the mean ± SEM. Two-tailed Student's t test followed by Tukey's post hoc test (3 independent replicates). (D) Quantification of (B). The scatter plots show the relative P-α-Syn level (normalized to the first group). ***P<0.001. The data are expressed as the mean ± SEM. Two-tailed Student's t test followed by Tukey's post hoc test (3 independent replicates). (E) Representative confocal 3D imaging of live cells transfected with LV-Lag3 or 3 shRNA alone or transfected with LV-Lag3 after 3 shRNA knockdown and then treated with Alexa Fluor 488-tagged α-Syn PFFs for 48 hours. (F) Quantification of (E). The scatter plots show the relative Alexa Fluor 488 fluorescent spot levels (normalized to the first group). *P<0.05, **P<0.01. The data are expressed as the mean ± SEM. One-way ANOVA with Bonferroni's post hoc test (3 independent replicates).

Fig. S7

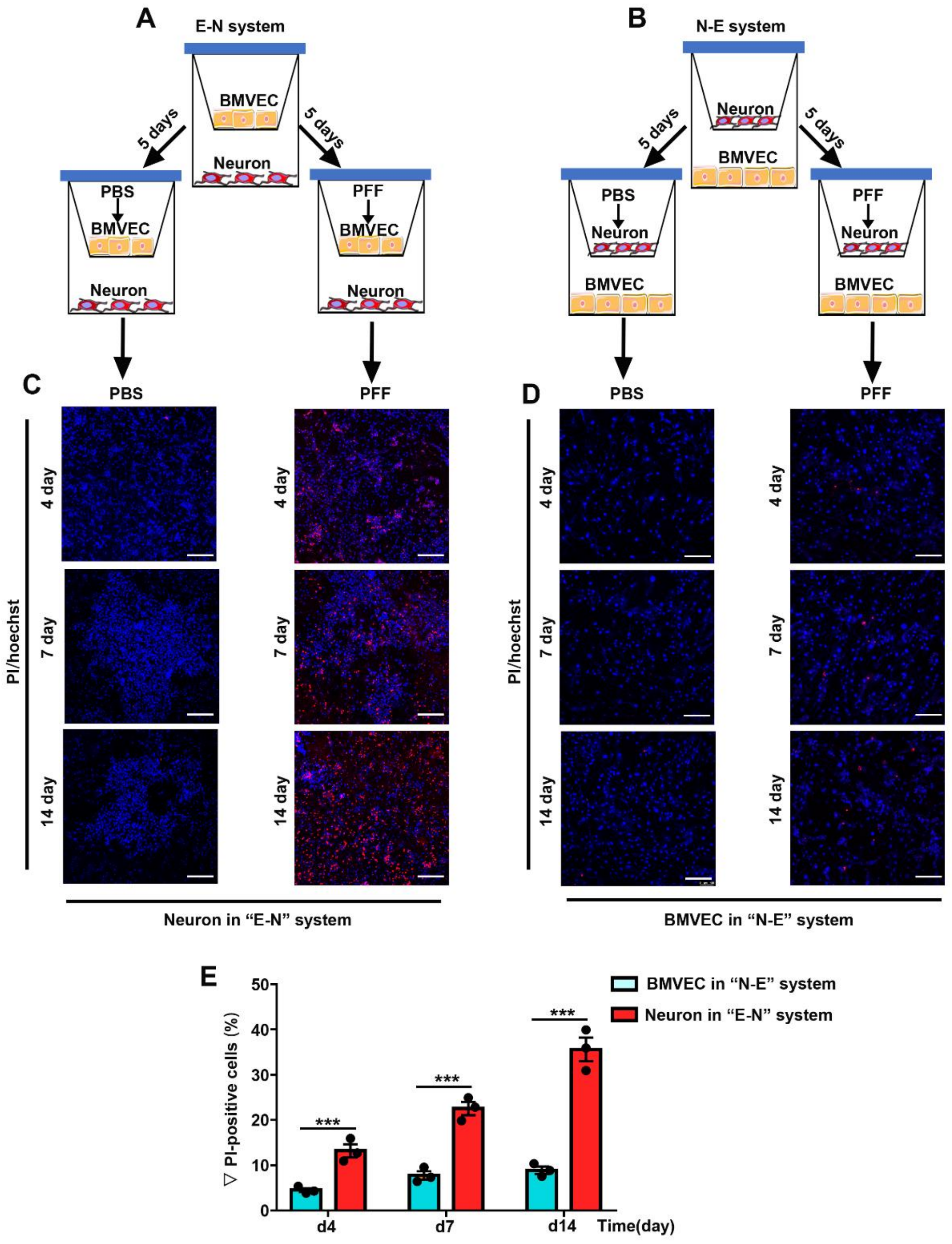

Fig. S7 Cell death rates in the ECs-to-neurons (E-N) and neurons-to-ECs (N-E) systems.

(A~B) Schematic of the experimental design of the ECs-to-Neurons (E-N) and neurons-to-ECs (N-E) systems. In the "E–N" system, primary mouse BMVECs were seeded on Transwell inserts (0.4 μm pore size), and primary cortical neurons were seeded in the

bottom of confocal plate (A); in the "N–E" system, neurons were seeded on Transwell inserts, and BMVECs were seeded in the bottom of confocal plate (B). After 5 days of cell seeding, the cells on Transwell inserts were treated with PBS or 5 μg/ml α-Syn PFFs, and cell death in the bottom of the confocal plate was assessed at 4, 7, and 14 days after PBS or PFFs treatment. The impact of EC α-Syn pathology on neurons or the impact of neuronal α-Syn pathology on ECs in the early, middle and late stages were reflected by the ▽cell death rate in the bottom of the two systems at 4, 7, and 14 days. The ▽cell death rate represented the cell death rate affected by PFFs, its value was the difference of the "PFFs treatment" minus the "PBS treatment". (C) Representative Hoechst and PI staining images of neurons in the bottom of confocal plate in the "E-N" system. The scale bar denotes 200 μm. (D) Representative Hoechst and PI staining images of BMVECs in the bottom of the confocal plate in the "N-E" system. The scale bar denotes 200 μm. (E) The statistical graph shows the ▽ PI-positive cells (▽ PI-positive cells = % of PI-positive cells of the "PFFs treatment" minus that of the "PBS treatment") of the "E-N" and "N-E" systems at time points of 4, 7, and 14 days, reflecting the▽cell death rate. ***$P<0.001$. The data are expressed as the mean ± SEM. Two-tailed Student's t test followed by Tukey's post hoc test (3 independent replicates).

Fig. S8

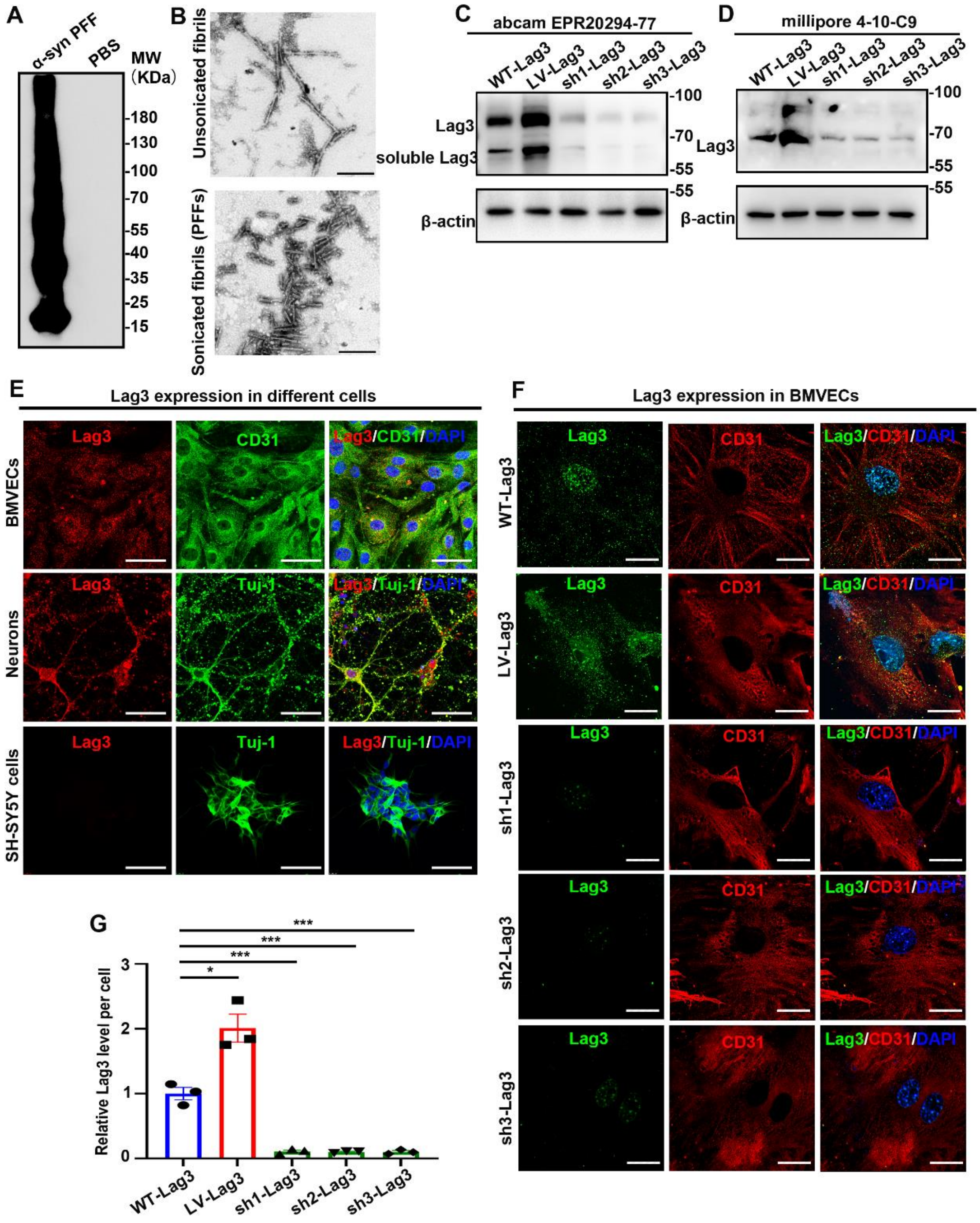

Fig. S8 Validation of α-Syn-PFFs, Lag3 antibody specificity and effect of LV-Lag3 or sh-Lag3. (A) Immunoblots for PBS or α-Syn PFFs indicating that α-Syn fibrils were successfully synthesized after 7 days of agitation. (B) Negative-stain TEM images showing the electronic fibril structure of α-Syn PFFs. α-Syn PFFs are a uniform specification of α-Syn fibrils with uniform sonication for experimental use. The length of α-Syn PFFs was controlled between 40~50 nm for cell and animal experiments. The

scale bar denotes 500 nm. (C~D) Immunoblots for LAG3 levels using the Lag3 antibody Millipore 410C9 (C) and Abcam EPR20294-77 (D) in primary mouse BMVECs transfected with LV-LAG3 lentivirus and 3 shRNAs targeting LAG3. (E) Immunofluorescence for Lag3 in primary neurons (as a positive control with Lag3 staining), SH-SY5Y cells (as a negative control without Lag3 staining) and primary BMVECs. (F) Immunofluorescence for Lag3 in primary mouse BMVECs transfected with LV-LAG3 lentivirus and 3 shRNAs targeting LAG3. (G) Quantification of (F). The scatter plots show the relative Lag3 level per cell. *P<0.05, ***P<0.001. The data are expressed as the mean ± SEM. One-way ANOVA by Bonferroni's post hoc test (3 independent replicates).

Fig. S9

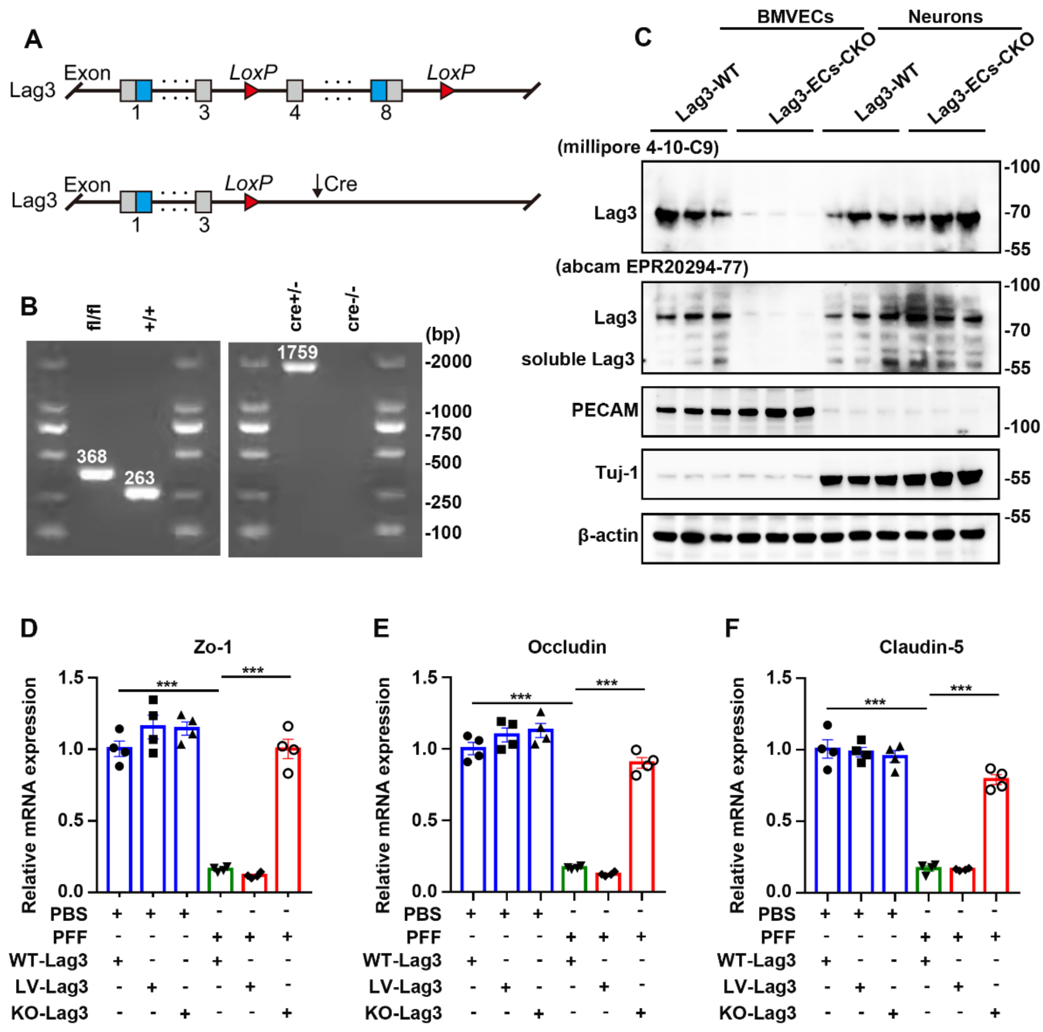

Fig. S9 Construction of Lag3-ECs-Cko mice and qRT–PCR analysis of tight junction proteins influenced by Lag3 in BMVECs. (A) Scheme of Lag3-ECs-Cko mouse generation. Loxp was placed before exon 4 and after exon 8. Expression of Cre leads to the deletion of exons 4-8. (B) PCR validation of mice with (fl/fl) or without (+/+) loxp insertion; with (+/-) or without (-/-) cre insertion. (C) Immunoblot validation of Lag3-ECs-Cko mice. The Lag3 antibodies Millipore 410C9 and Abcam EPR20294-77 were used to verify whether Lag3 was specifically knocked out in endothelial tissue but not in neuronal tissue. PECAM and TUJ-1 antibodies were used in combination to detect the purity of isolated brain microvascular tissue (PECAM, specifically expressed in BMVECs) or neuronal tissue (TUJ-1, specifically expressed in neurons). (D~F) qRT–PCR analysis of the relative mRNA expression of ZO-1 (D), Occludin (E), and Claudin 5 (F). The cells were primary BMVECs extracted from WT or $Lag3^{-/-}$ mice or LV-Lag3-transfected WT mouse BMVECs (cells from $Lag3^{-/-}$ mice and WT mice were transfected with negative control lentivirus) treated with PBS or 5 μg/ml α-Syn PFFs for 14 days. ***P<0.001. The data are expressed as the mean ± SEM. Two-tailed Student's t test followed by Tukey's post hoc test (4 independent replicates).

Fig. S10

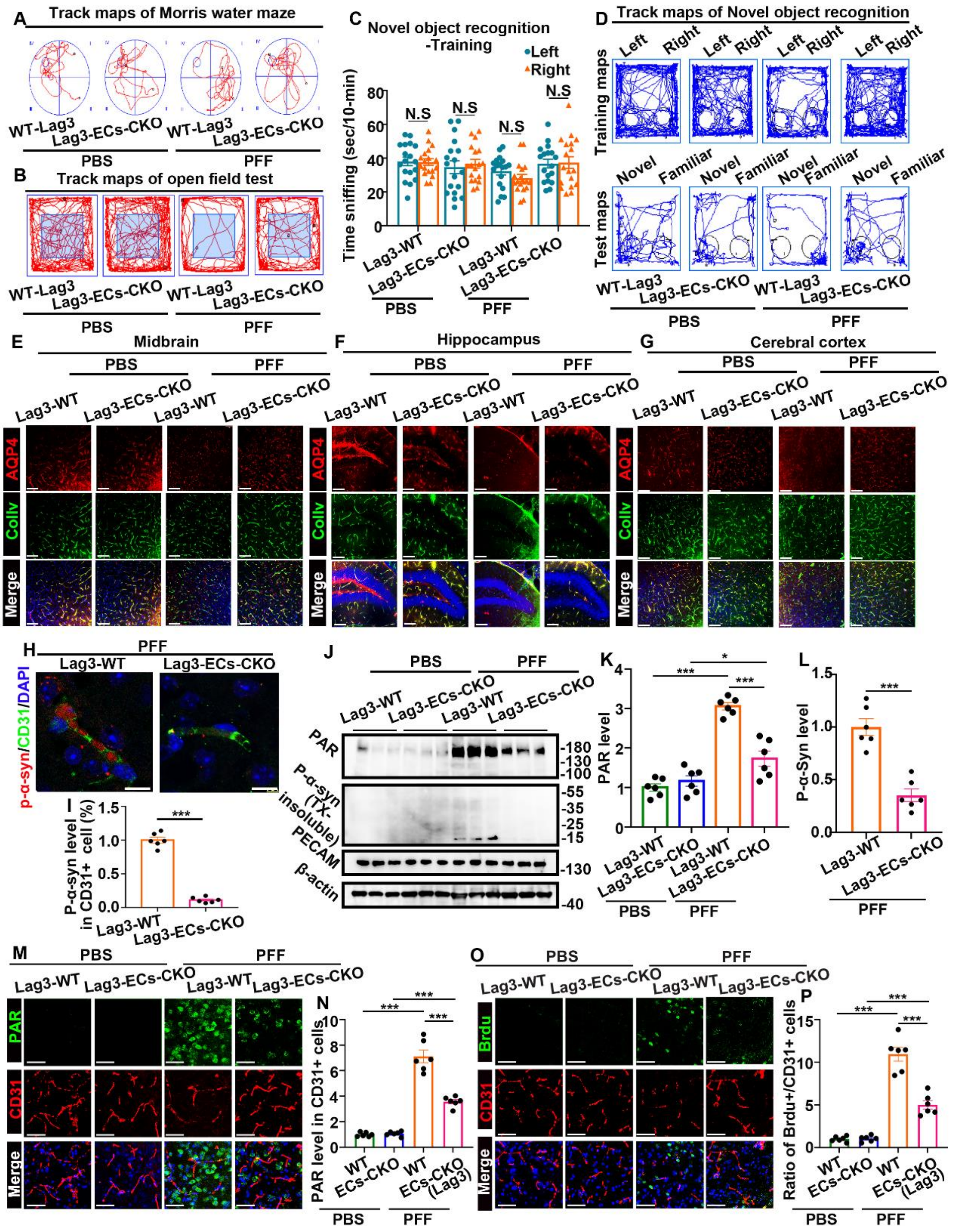

Fig. S10 Influence of cognition and the cerebral microvascular system by specific knockout of Lag3 in BMVECs. Mice were grouped into PBS-injected wild-type mice (PBS-Lag3-WT), PBS-injected Lag3-ECs-Cko mice (PBS-Lag3-ECs-Cko), PFF-injected wild-type mice (PFF-Lag3-WT) and PFF-injected Lag3-ECs-Cko mice (PFF-Lag3-ECs-Cko). Six months post-injection was set as the time point to detect cognitive

and cerebral microvascular indicators. (A) Representative track maps from the MWM. (B) Representative track maps from the open field test. (C~D) NOR test: (C) Scatter plots showing the time spent sniffing the left or right object for 10 minutes in the training period, N.S means no significance (n=18). (D) Representative track maps from the NOR test. (E~G) Representative immunofluorescence costaining images of AQP4, collagen IV and merge (AQP4/ColIV/DAPI) in the midbrain (E), hippocampus (F), and cerebral cortex (G). The scale bar denotes 100 μm. (H) Representative images of P-α-Syn/CD31/DAPI. The scale bar denotes 20 μm. (I) Quantification of (H). The scatter plots show the relative P-α-Syn level in $CD31^+$ cells of PFF-Lag3-WT or PFF-Lag3-ECs-Cko mice. ***$P<0.001$ (n=6). (J) Representative western blot imaging of PAR and p-α-Syn (TX-insoluble) in isolated brain microvascular tissue. (K~L) Quantification of (J). The scatter plots show the relative PAR level (K) and TX-insoluble p-α-Syn level (L). (M) Representative immunofluorescence costaining images of PAR and CD31. (N) Quantification of (J). The scatter plots show the relative PAR level in $CD31^+$ cells. (O) Representative immunofluorescence costaining images of BrdU and CD31. (P) Quantification of (O). The scatter plots show the relative ratio of $BrdU1^+/CD31^+$ cells. All data are expressed as the mean ± SEM. Differences among multiple means, one-way ANOVA by Bonferroni's post hoc test; differences between 2 means, two-tailed Student's t test followed by Tukey's post hoc test.

Supplementary Table 1

Summary of changes in capillary ultrastructure evaluated by TEM

	Sham /	AAV/2 S-1	AAV/2 S-2	AAV/2 S-3	PFF S-1	PFF S-2	PFF S-3
BM							
	86.67	55.67	63.67	66.67	30.33	41.67	48.33
Intact	±3.40	±3.30	±2.05	±2.49	±2.49	±5.31	±4.47
Increased	11.67	32.67	28.67	26.67	46.33	42.33	40.33
thickness	±2.36	±2.49	±2.30	±1.70	±3.09	±3.30	±2.62
Detachment	1.67	11.67	7.67	6.67	23.33	15.67	11.33
	±1.25	±2.87	±2.36	±2.05	±1.70	±2.05	±1.89
TJ							
Intact	81.67	53.33	61.67	65.33	46.33	51.33	58.67
	±4.99	±5.31	±5.79	±5.91	±6.13	±7.04	±6.13
Discontinuous	18.33	44.67	38.33	34.67	53.33	48.67	41.33
	±4.99	±5.31	±5.79	±5.91	±6.13	±7.04	±6.13

The values are shown as the percentages of different forms of the basement membrane (BM) and tight junctions (TJs), and the results are expressed as the mean ± SEM. The are statistically significant differences (***$P<0.001$) among the means of the sham, AAV and PFF groups in S-1, S-2 and S-3. One-way ANOVA with Bonferroni's post hoc test.

Supplementary Table 2

Primer sequences

RT–PCR	Forward	Reverse
ACTB	GGCTGTATTCCCCTCCATCG	TCCCCACTCTGAAAATGAGGA
Zo-1	GCCGCTAAGAGCACAGCAA	GGCTCCTGCCTACAAGAGTG

Occludin	TTGAAAGTCCACCTCCTTACAGA	CCGGATAAAAAGAGTACGCTGG
Claudin-5	GCAAGGTGTATGAATCTGTGCT	GTCAAGGTAACAAAGAGTGCCA

Supplementary Table 3

List of antibodies used in this study

Antibodies	Source/Cat. No./Ref.	Host	Dilution
Tyrosine Hydroxylase (TH)	Millipore (AB152)	Rabbit	1:800 (IHC/IF)
NeuN	Abcam (ab177487)	Rabbit	1:1000 (IHC)
CD34	Santa Cruz (sc-7324)	Mouse	1:200 (IF)
IBA1	Abcam (ab283319)	Mouse	1:400 (IF)
GFAP	Abcam (ab7260)	Rabbit	1:400 (IF)
AQP4	Abcam (ab9512)	Mouse	1:200 (IF)
Collagen IV	Abcam (ab6585)	Rabbit	1:200 (IF)
α-Syn	Abcam (ab212184)	Rabbit	1:1000 (WB)
p-α-Syn (Ser129)	Abcam (ab 51253)	Rabbit	1:500 (WB)<br>1:100 (IF)
β-actin	Cell Signaling (4970)	Rabbit	1:1000 (WB)
ZO-1	Abcam (ab96594)	Rabbit	1:1000 (WB)
Occludin	Abcam (ab167161)	Rabbit	1:1000 (WB)
Claudin-5	Abcam (ab131259)	Rabbit	1:1000 (WB)
Rab7	Cell Signaling (2094)	Rabbit	1:1000 (WB)
PECAM (CD31)	Cell Signaling (3528)<br>Millipore (SAB5700639)<br>Abcam (ab24590)	Mouse<br>Rabbit<br>Mouse	1:1000 (WB)<br>1:100 (IF)
Tuj-1	Proteintech (66375)	Mouse	1:3000 (WB)<br>1:500 (IF)

PAR	Santa Cruz (sc-56198)	Mouse	1:200 (WB)<br>1:50 (IF)
γH2A.X	Abcam (ab81299)	Rabbit	1:5000 (WB)<br>1:500 (IF)
Lag3	Abcam (209238, EPR20294)<br>Millipore (MABF954,4-10-C9)	Rabbit<br>Mouse	1:1000 (WB)<br>1:300 (IF)
α-SMA	Proteintech (67735)	Mouse	1:300 (IF)
NG2	Santa Cruz (sc-53389)	Mouse	1:150 (IF)

Supplementary Table 4

Sequence information

**shRNA**	**Sense sequence (5’-3’)**
shRNA1-LAG3	CACCACTTAGCGGAAAGCTTCCTCT
shRNA2-LAG3	GCTGCTTTGTGAGGTGACTCCAGTA
shRNA3-LAG3	CACCTCCTGCTGTTTCTCATCCTTG
